# Supplementary material for: How do musculoskeletal disorders impact on quality of life in Tanzania? Results from a community-based survey
Source: BMJ Open. 2025 Dec 1;15(12):e092877. doi: 10.1136/bmjopen-2024-092877 (PMC12706106; doi:10.1136/bmjopen-2024-092877)
Supplement: Supplementary data [file bmjopen-15-12-s001.pdf]

Supplementary 1: Model fit tests

GLMDIAGL: This program runs the modified Park test, Pearson correlation test, Pregibon link test, and modified Hosmer and Lemeshow test, for a set of power links that range between -2 and 2 in intervals of 0.1. It records the results of these tests as well as the log likelihood, AIC, BIC statistics from each power model. Saved statistics are for the model that uses the family specified by the modified Park test. [glmdiagl "glm QoLUg i.rems_ctrl age i.sex i.religion i.edusimple i.labour i.marrydummy i.familypain i.intsmoke i.intdrink i.everpregnant i.diabetest3 i.hypertensiont3 i.MM, vce(cluster clustervar)"]

```
                _______________ Summary Score Recommendations________________
                 3-(sum p)      sum abs|log(p)|    3-(sum p^.5)      sum(1-p)^2
Ranking         Link   Family    Link   Family    Link   Family    Link   Family

Preferred       1.00      0      -2.00     0      1.00      0      1.00      0
2nd best        0.90      0      -1.90     0      0.90      0      0.90      0
3rd best        0.80      0      -1.80     0      0.80      0      0.80      0

See r(linkmat1), r(linkmat2), and r(familymat) for more detailed results

Family Coding (for r(familymat)

0 = gaussian; 1 = poisson; 2 = gamma; 3 =igaussian; 4 = Preferred family switches
''Conflicting families'': 40 = gaussian; 41 = poisson; 42 = gamma; 43 = igaussian
''Initial values infeasible'': 70 = gaussian; 71 = poisson; 72 = gamma; 73 = igaussian;
5 = None
```

Source: https://drhenryglick.com/identifying-better-links-and-families/

```
 FITTED MODEL:  Link = Power  1; Family =  Gaussian

 Results, Modified Park Test (for Family)

    Coefficient:   -2.60235

    Family, Chi2, and p-value in descending order of likelihood

       Family                          Chi2        P-value

       Gaussian NLLS:               86.6945         0.0000
       Poisson:                    166.1237         0.0000
       Gamma:                      271.1560         0.0000
       Inverse Gaussian or Wald:   401.7912         0.0000


 Results of tests of GLM Power  1 link

       Pearson Correlation Test:                    1.0000
       Pregibon Link Test:                          0.0006
       Modified Hosmer and Lemeshow:                0.0000
       SS: sqr1_p                                   1.9988
       SS: sqrtp                                    1.9755
       SS: logp                                    32.7470
       SS: sump                                     1.9994
       R-square                                     0.3478
       Log likelihood                             -52.9043
       AIC                                          0.2865
       BIC                                      -2880.1483
       Deviance                                    35.4903


 **Gaussian family given identity link
```
