# Supplementary material for: How do musculoskeletal disorders impact on quality of life in Tanzania? Results from a community-based survey
Source: BMJ Open. 2025 Dec 1;15(12):e092877. doi: 10.1136/bmjopen-2024-092877 (PMC12706106; doi:10.1136/bmjopen-2024-092877)

Supplementary 2: Distributions of Utilities

Distribution of utilities using Ugandan and Ethiopian tariffs: population

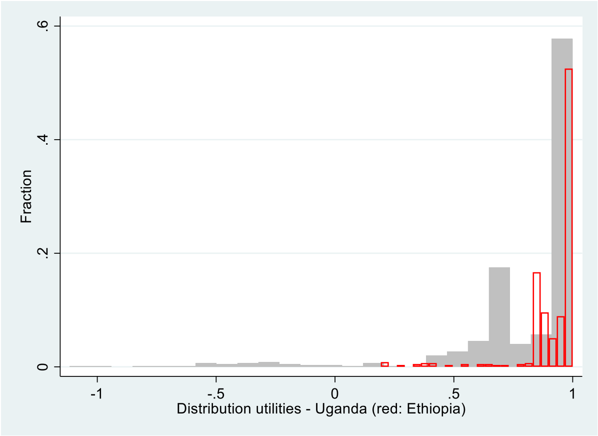

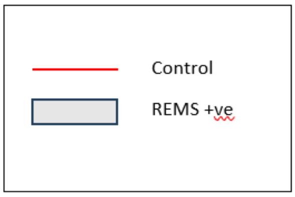

Distribution of utilities using Ugandan and Ethiopian tariffs: REMS positive

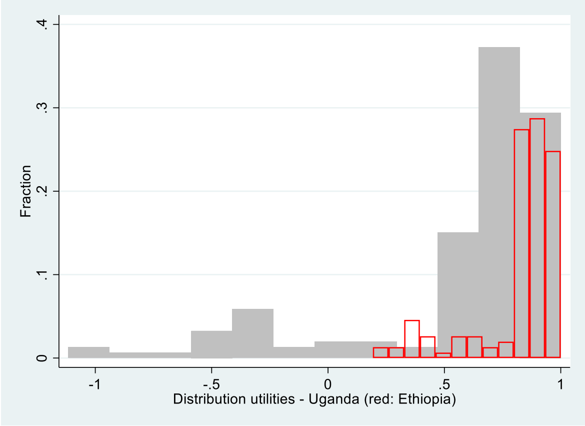

Distribution of utilities using Ugandan and Ethiopian tariffs: Controls

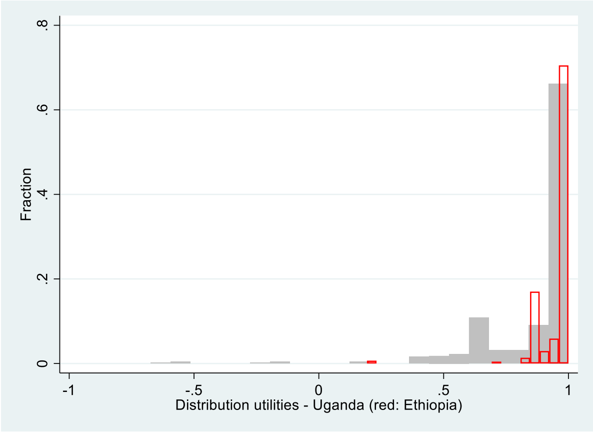

Supplement: Supplementary data [file bmjopen-15-12-s002.pdf]
