# Supplementary material for: How do musculoskeletal disorders impact on quality of life in Tanzania? Results from a community-based survey
Source: BMJ Open. 2025 Dec 1;15(12):e092877. doi: 10.1136/bmjopen-2024-092877 (PMC12706106; doi:10.1136/bmjopen-2024-092877)
Supplement: Author's manuscript [file bmjopen-2024-092877.draft_revisions.pdf]

BMJ Open is committed to open peer review. As part of this commitment we make the peer review history of every article we publish publicly available.

When an article is published we post the peer reviewers' comments and the authors' responses online. We also post the versions of the paper that were used during peer review. These are the versions that the peer review comments apply to.

The versions of the paper that follow are the versions that were submitted during the peer review process. They are not the versions of record or the final published versions. They should not be cited or distributed as the published version of this manuscript.

BMJ Open is an open access journal and the full, final, typeset and author-corrected version of record of the manuscript is available on our site with no access controls, subscription charges or pay-per-view fees (http://bmjopen.bmj.com).

If you have any questions on BMJ Open's open peer review process please email
info.bmjopen@bmj.com

BMJ Open

How do Musculoskeletal Disorders impact on Quality of Life in Tanzania? Results from a Community-Based Survey

Journal:	*BMJ Open*
Manuscript ID	bmjopen-2024-092877
Article Type:	Original research
Date Submitted by the Author:	26-Aug-2024
Complete List of Authors:	Grieve, Eleanor; University of Glasgow College of Medical Veterinary and Life Sciences,<br>Deidda, Manuela; University of Glasgow, Health Economics and Health Technology assessment<br>Krauth, Stefanie; University of Glasgow; University of Glasgow<br>Biswaro, Sanjura Mandela; Kilimanjaro Christian Medical Center<br>Halliday, J; University of Glasgow<br>Hsieh, Ping-Hsuan; National Defense Medical Center<br>Kelly, Clive; James Cook University Hospital; Newcastle University<br>Kilonzo, Kajiru; Kilimanjaro Christian Medical University College, Medicine<br>Kiula, Kiula P.; Kilimanjaro Christian Medical Center<br>Kolimba, Rose; Kilimanjaro Christian Medical Center<br>Msoka, Elizabeth ; Kilimanjaro Christian Medical University College, Clinical Trial<br>Siebert, Stefan; University of Glasgow<br>Walker, Richard William; North Tyneside General Hospital<br>Yongolo, Nateiya; Kilimanjaro Christian Medical Center<br>Mmbaga, Blandina T.; Kilimanjaro Christian Medical Center; Kilimanjaro Christian Medical University College<br>McIntosh, Emma; University of Glasgow
Keywords:	Musculoskeletal disorders < ORTHOPAEDIC & TRAUMA SURGERY, HEALTH ECONOMICS, Quality of Life, Cross-Sectional Studies

SCHOLARONE™
Manuscripts

I, the Submitting Author has the right to grant and does grant on behalf of all authors of the Work (as defined in the below author licence), an exclusive licence and/or a non-exclusive licence for contributions from authors who are: i) UK Crown employees; ii) where BMJ has agreed a CC-BY licence shall apply, and/or iii) in accordance with the terms applicable for US Federal Government officers or employees acting as part of their official duties; on a worldwide, perpetual, irrevocable, royalty-free basis to BMJ Publishing Group Ltd ("BMJ") its licensees and where the relevant Journal is co-owned by BMJ to the co-owners of the Journal, to publish the Work in this journal and any other BMJ products and to exploit all rights, as set out in our licence.

The Submitting Author accepts and understands that any supply made under these terms is made by BMJ to the Submitting Author unless you are acting as an employee on behalf of your employer or a postgraduate student of an affiliated institution which is paying any applicable article publishing charge ("APC") for Open Access articles. Where the Submitting Author wishes to make the Work available on an Open Access basis (and intends to pay the relevant APC), the terms of reuse of such Open Access shall be governed by a Creative Commons licence – details of these licences and which Creative Commons licence will apply to this Work are set out in our licence referred to above.

Other than as permitted in any relevant BMJ Author's Self Archiving Policies, I confirm this Work has not been accepted for publication elsewhere, is not being considered for publication elsewhere and does not duplicate material already published. I confirm all authors consent to publication of this Work and authorise the granting of this licence.

For peer review only

How do Musculoskeletal Disorders impact on Quality of Life in Tanzania? Results from a Community-Based Survey

Authors: Eleanor Grieve[1,◊], Manuela Deidda[1], Stefanie J Krauth[1,2], Sanjura M Biswaro[3], Jo E. B. Halliday[2], Ping-Hsuan Hsieh[5], Clive Kelly[6,7], Kajiru Gad Kilonzo[3], Kiula P Kiula[3], Rose Kolimba[3], Elizabeth F. Msoka[3], Stefan Siebert[8], Richard Walker[9], Nateiya Yongolo[3], Blandina T. Mmbaga[3,4], Emma McIntosh[1] and the NIHR Global Health Group

[1] School of Health and Wellbeing, College of Medical, Veterinary and Life Sciences, University of Glasgow, Clarice Pears Building, Glasgow, G12 8TB, Scotland
[2] School of Biodiversity, One Health and Veterinary Medicine, College of Medical, Veterinary and Life Sciences, University of Glasgow, G12 8QQ, Glasgow, UK
[3] Kilimanjaro Clinical Research Institute, Kilimanjaro Christian Medical Center, Sokoine Road Box 2236, Moshi, United Republic of Tanzania
[4] Kilimanjaro Christian Medical University College, Sokoine Road Box 2240 Moshi, Tanzania
[5] School of Pharmacy, National Defense Medical Center, Taipei, Taiwan
[6] James Cook University Hospital, Middlesbrough, Marton Rd, Middlesbrough TS4 3BW, England
[7] Newcastle University, Claremont Road, Newcastle upon Tyne NE1 7RU, England
[8] School of Infection and Immunity, College of Medical, Veterinary and Life Sciences, University of Glasgow, Sir Graham Davies Building, 120 University Place, Glasgow G12 8QQ, Scotland
[9] Northumbria Healthcare NHS Foundation Trust, North Tyneside General Hospital, North Shields, England

[◊] Corresponding author: Eleanor Grieve, School of Health and Wellbeing, University of Glasgow, Clarice Pears Building, Glasgow, G12 8RZ, Scotland
E-mail: Eleanor.Grieve@glasgow.ac.uk
Access to the data can be requested through the corresponding author

NIHR Global Health Research Group on Arthritis in Tanzania: nihr-ghrg-arthritis@glasgow.ac.uk

Keywords: Arthritis, joint disorders, musculoskeletal disorders, Tanzania, cross-sectional, health related quality of life, EQ5D

Word count: 3286

For peer review only - http://bmjopen.bmj.com/site/about/guidelines.xhtml

Article Summary

Abstract

Objectives

There are little available data on the prevalence, economic and quality of life impacts of musculoskeletal disorders in Sub-Saharan Africa (SSA). This lack of evidence is wholly disproportionate to the significant disability burden of musculoskeletal disorders as reported in high-income countries. Our research aimed to undertake an adequately powered study to identify, measure and value the health impact of musculoskeletal conditions in the Kilimanjaro region, Tanzania.

Design

A community-based cross-sectional survey was undertaken between January 2021 and September 2021. A two-stage cluster sampling with replacement and probability proportional to size was used to select a representative sample of the population.

Setting

The survey was conducted in 15 villages in the Hai District, Kilimanjaro region, Tanzania.

Participants

Economic and health-related quality of life (HRQOL) questionnaires were administered to a sample of residents (aged over 5 years old) in selected household (N = 1050). There was a total of 594 respondents, of whom 153 had a confirmed musculoskeletal disorder and 441 matched controls. Almost three quarters of those identified as having a musculoskeletal disorder were female and had an average age of 66 years.

Primary and secondary outcome measures

Questions on health care resource use, expenditure and quality of life were administered to all participants, with additional more detailed economic and quality of life questions administered to those who screened positive, indicating probable arthritis.

Results

There is a statistically significant reduction in HRQOL, on average 25% from a utility score of 0.86 (0.837, 0.885) to 0.64 (0.582, 0.693) for those identified as having a musculoskeletal disorder compared to those without. The attributes 'pain' and 'discomfort' were the major contributors to this reduction in HRQOL.

Conclusions

This research has revealed a significant impact of musculoskeletal conditions on HRQOL in the Hai district in Tanzania. The evidence will be used to guide clinical health practices, interventions design, service provisions, and health promotion and awareness activities at institutional, regional and national levels.

Strengths and limitations of this study

- This is the first powered study of its kind in musculoskeletal disorders in Tanzania (and likely wider SSA) using valid screening tools along with estimates of preference-based quality of life.
- This study has revealed and quantified the significant health impact associated with musculoskeletal conditions in the Hai District, Kilimanjaro region, Tanzania.
- A limitation of this study is that Tanzania has no value set for utilities. Instead, we used the weights generated in neighbouring Uganda to value health profiles and undertook a sensitivity analysis using the value sets from Ethiopia.

For peer review only

Background

Musculoskeletal disorders are ranked as the second most common cause of disability worldwide(1). These conditions cause not only clinical but economic, societal, and quality of life impacts on people's day-to-day lives. Furthermore, musculoskeletal conditions often predispose and directly contribute to other comorbidities and non-communicable diseases (NCDs). Epidemiological studies confirm the strong relationship between painful musculoskeletal conditions, lack of physical activity and resulting functional decline with frailty, loss of wellbeing and loss of independence and depressive symptoms (2, 3). Musculoskeletal health is critical for people's mobility and dexterity, their ability to work and actively participate in all aspects of life and to maintain economic, social and functional independence across their life course (4). Musculoskeletal conditions have suffered from a distinct lack of attention from the medical profession, politicians, and the public because they are not usually life threatening and many are considered to be a natural part of aging, despite being the most prevalent causes of disability, absence from occupational activities and early retirement impacting both the individual and also wider society (5).

However, little evidence exists on the prevalence, patterns and impacts of such conditions among the general population in Sub-Saharan Africa (SSA) (4). Further, existing strategic plans to reduce NCDs in this setting often overlook the impact of musculoskeletal disorders. When prioritising healthcare research and investment, the burden of musculoskeletal disorders has been underestimated and even ignored, mainly due to their chronic nature and perceived low fatality rate. Whilst the importance and severity of the emerging NCD burden is well recognised with strategic national action plans in place for the prevention and control of NCDs, the focus is dominated by conditions such as cardiovascular diseases, cancer and diabetes. Little attention has been paid to musculoskeletal conditions. This is out of proportion to the impact that musculoskeletal conditions have on disability, with musculoskeletal being one of the major causes of non-traumatic disability within the global burden of disease (6). Arthritis (joint diseases)are among the commonest musculoskeletal disorders and are a leading cause of physical disability and severe long-term pain. Measuring the full impact of these disorders is urgently needed to inform policy.

Tanzania has made progressive commitments towards Universal Health Coverage (UHC) with the scale up of health insurance to be rolled out in 2023 and has launched a National NCDs strategic plan. However, plans often prioritise other NCDs conditions such as diabetes, hypertension and sickle cell disease whilst leaving musculoskeletal conditions behind. The 'NIHR Global Health Research Group on estimating the prevalence, quality of life, economic and societal impact of arthritis in Tanzania: a mixed methods study at University of Glasgow'[1] was established with the aim of characterising and estimating the various burdens of arthritis in Tanzania. This wide-ranging study aimed to establish the prevalence of arthritis and joint problems and, to better understand their impact - clinically, economically and socially - in Tanzania. Here, we present the findings related to the impact on health-related quality of life (HRQOL) for those with musculoskeletal conditions and arthritis in Tanzania.

Methods

A community-based cross-sectional survey conducted in 15 villages in the Hai District, Kilimanjaro was undertaken between January 2021 and September 2021 working in partnership with the Kilimanjaro Clinical Research Institute (KCRI). Clinical screening tools, including the Gait Arms Legs Spine (GALS) (7) and Regional Examination of the Musculoskeletal system (REMS) (8), were used to screen people for musculoskeletal conditions through a tiered approach. A two-stage cluster sampling with

[1] https://fundingawards.nihr.ac.uk/award/17/63/35

For peer review only - http://bmjopen.bmj.com/site/about/guidelines.xhtml

replacement and probability proportional to size was used to select a representative sample of the population(9). We administered a battery of instruments including economic (i.e. usage of healthcare resources; productivity impact etc.) and HRQOL questions to a sample of residents (aged over 5 years old) in selected household (N = 1050 households). Questions on health care resource use, expenditure and quality of life were administered to all participants, with additional more detailed (long version) economic and quality of life questions administered to those who screened positive on REMS (only GALS positive individuals were assessed with REMS), indicating probable arthritis. In order to establish a reference population for these measures, this group were matched on age (+/- 1 year for minors and +/-3 years for adults)[2] and gender, aiming for 2 controls per REMS positive case from the population of GALS negative participants. Hence responses from participants screening positive for musculoskeletal conditions were able to be directly compared to a matched control group in a bid to establish the relative magnitude of quality of life impact of the musculoskeletal disorders. Full details are provided in the study protocol (10).

Quality of life tools

The Swahili version of the preference-based tool EuroQol (EQ-5D-5L) was used for those 18 years and over(11). The EQ-5D is a generic questionnaire relating to mobility, self-care, usual activity, pain and discomfort, and anxiety and depression. The EQ-5D health state is converted into a utility score (typically between 0-1 where 0 represents death and 1 represents full health) using a country-specific scoring algorithm or value set. As Tanzania has no set of weights for the EQ-5D dimensions, the Ugandan tariff was used as the base case and the Ethiopian tariff as a sensitivity analysis(12, 13).The Child Health Utility (CHU9D) instrument was used for children and adolescents under 18 years (14).

Model

A generalised linear model (GLM) model with appropriate family/link was fitted to estimate differences in utility scores (dependent variable) between those presenting as REMS positive i.e. with probable musculoskeletal disorder and controls. The modified Park test was conducted to choose the best distribution while a suite of tests was run to guide the best choice of link. See Supplementary File 1 for full details. Explanatory variables in the model included age, occupation, marital status, gender, religion, education, co-morbidities and behavioural risk factors. Although a matched control group was established, we still controlled for sex and age in our case control model to insure against imperfect matching (15). Standard errors were adjusted for clustering at household and village level. Multiple imputation procedures using chained equations were used to impute missing data, creating 40 imputed datasets (16). Both complete case and multiple imputation analyses were undertaken. All analyses were performed using Stata version 17.

Patient and public involvement

The community were involved throughout this research, from design to dissemination. Please see report which details the dissemination outputs, stakeholder engagement activities and their underlying principles conducted by this study (17).

Ethical considerations

The study was conducted in accordance with national and international ethical guidelines and principles and received clearance from the College of Medical, Veterinary & Life Sciences Ethics Committee at the University of Glasgow (Ref. number: 200180100), the Kilimanjaro Christian Medical

[2] In cases where no age match could be identified within this range, e.g. for very old participants, larger age-differences were accepted rather than not recruiting a control.

For peer review only - http://bmjopen.bmj.com/site/about/guidelines.xhtml

University College Local Ethical Review Committee (KCMC/P.I/Vol.XI), and the National Institute for Medical Research in Tanzania (NIMR/HQ.R.8a/Vol.IX/3038). Written informed consent was obtained from all adult participants and guardians of participants aged <18 years after detailed information about the study was provided and time allowed for any questions. In addition to guardian consent, assent was obtained from participants aged 12-17 years. Consent documents were all provided in Swahili. In case of illiteracy or other language requirements, a witness and/or translator could be chosen by the participants to provide verbal translation during consenting and survey delivery. All data collection instruments were translated to Swahili and adapted to the local context(18, 19).

Results

There was a total of 594 respondents, of whom 153 had a confirmed musculoskeletal disorder and 441 matched controls. Table 1 provides a descriptive analysis comparing REMS positive individuals and matched controls, in terms of demographic, clinical and socio-economic characteristics. Almost three quarters of those identified as REMS positive were female and had an average age of 66 years. They were twice as likely to not be working than those without a musculoskeletal disorder (12% compared with 6%). They were also more likely to have other conditions, in particular, hypertension (24% compared with 14%). Quality of life utility scores by age and count of co-morbidities showed a clear distinction between those with a musculoskeletal disorder, compared to those without a musculoskeletal disorder for all age groups.

In terms of missing data, although only one person did not answer the EQ-5D questionnaire, a 18% rate of missingness in the covariates (especially history of family pain) required the use a of a multiple imputation approach. Two children were positive with GALS, one refused to be examined further with REMS, and the other was REMS positive.

For peer review only - http://bmjopen.bmj.com/site/about/guidelines.xhtml

Table 1: Descriptive summary of characteristics for all participants, and by musculoskeletal /arthritis positive and matched controls.

Categories	All (n = 594)	REMS+ (n = 153)	Controls (n = 441)
Background characteristics			
**Age (mean)**	62.8	66.4	61.5
**Gender (female)**	70%	74%	68%
**Occupation (farmer)**	78%	78%	78%
**Unemployed or retired**	7.24%	11.8%	5.67%
**Religion**			
Christian	82%	79%	84%
Muslim	18%	21%	16%
**Education**			
Basic	87%	91%	86%
Middle	9%	8%	10%
Higher	4%	1%	4%
**Married**	58%	49%	61%
**Smoker**			
Current	7%	5%	8%
Former	9%	10%	9%
Never	84%	85%	83%
**Alcohol Drinker**			
Current	31%	32%	31%
Former	23%	21%	24%
Never	46%	47%	45%
**History of family pain**	9%	25%	3%
Other conditions			
+1 any other condition (n)	18% (96)	26% (39)	15% (57)
+2 any other conditions (n)	3% (18)	3% (5)	3% (13)
Hypertension (n)	17% (89)	24% (37)	14% (52)
Diabetes (n)	5% (29)	5% (8)	6% (21)
Tuberculosis (n)	2% (8)	1% (2)	2% (6)
Quality of life (Utility weight (0-1)[3](20)			
**All**	0.80 (594)	0.57 (153)	0.88 (441)
**By age:**			
< 30 years (n)	0.94 (15)	0.61 (2)	0.99 (13)
30 – 40 years (n)	0.91 (14)	1.0 (2)	0.90 (12)
40-50 years (n)	0.93 (56)	0.84 (15)	0.96 (41)
50 – 60 years (n)	0.87 (136)	0.73 (29)	0.91 (107)
60 – 70 years (n)	0.83 (191)	0.64 (43)	0.89 (148)
> 70 years (n)	0.67 (182)	0.37 (62)	0.82 (120)
**By comorbidity:**			
+1 condition (n)	0.75 (96)	0.62 (39)	0.85 (57)
+2 conditions (n)	0.68 (18)	0.32 (5)	0.82 (13)

[3] The value attached to an EQ-5D profile according to a set of weights that reflect, on average, people's preferences about how good or bad the state is. Values are anchored at 1 (full health) and 0 (a state as bad as being dead) as required by their use in economic evaluation.

For peer review only - http://bmjopen.bmj.com/site/about/guidelines.xhtml

Table 2 provides the results for both the complete case and multiple imputed datasets using the Ugandan and Ethiopian tariffs. Using the chosen family of Gaussian non-linear least squares with identity link and taking the Ugandan results as the base case, there is a statistically significant reduction in EQ-5D HRQOL, on average 25% from a utility score of 0.8 to 0.6, for those identified as having a musculoskeletal disorder compared to those without. Age, history of family pain and no longer working are associated with a statistically significant reduction in quality of life in both the complete case and multiple imputed analyses. Gender was not a significant factor. Whilst women are more likely to have a musculoskeletal disorder, there was no significant difference between men and women in terms of reduced HRQOL. Comorbidities do not have a statistically significant impact on HRQOL. This is likely due to the relatively low number of co-morbidities in our data.

Using the Ethiopian value set for the purposes of sensitivity analyses, there is again a statistically significant reduction in quality of life, on average of around 10% from a utility score of 0.9 to 0.8, for those identified as REMS positive compared with those who are not. Absolute utility values across both groups are higher using the Ethiopian value set as compared with those generated by the Ugandan value set. Age, history of family pain and no longer working are similarly associated with a statistically significant reduction in quality of life in both the complete case and multiple imputed analyses.

All five dimensions of the EQ-5D - mobility, self-care, usual activity, pain and discomfort, and anxiety and depression - were scored lower by those with a positive diagnosis. Notably, 'pain and discomfort' was a major contributor to this reduction in quality of life. The distribution of each HRQOL dimension (for the Ugandan tariff only) is shown in Figure 1. The controls (in red) show a similar distribution across each domain skewed towards the scores indicating 'none' or 'mild' problems, in contrast the pattern for those with musculoskeletal indicates greater numbers scoring moderate and severe problems in each domain.

For peer review only

For peer review only - http://bmjopen.bmj.com/site/about/guidelines.xhtml

Table 2: EQ-5D scores and associated decrements for those identified positive as having a musculoskeletal disorder compared with matched controls – complete case and multiple imputation using Ugandan (base case) and Ethiopian (sensitivity analysis) tariffs.

	Ugandan tariff				Ethiopian tariff			
Categories	Quality of life decrements (Complete case) N = 488		Quality of life decrements (Multiple imputation) N = 594		Quality of life decrements (Complete case) N = 488		Quality of life decrements (Multiple imputation) N = 594	
	QoL (95% CI)	p-value	QoL (95% CI)	p-value	QoL (95% CI)	p-value	QoL (95% CI)	p-value
MSK[4] (REMS+)	-0.226 (-0.296, -0.157)	0.000	-0.224 (-0.286, -0.162)	0.000	-0.094 (-0.123, -0.065)	0.000	-0.095 (-0.121, -0.068)	0.000
Age	-0.005 (-0.007, -0.003)	0.000	-0.005 (-0.007, -0.003)	0.000	-0.002 (-0.003, -0.001)	0.000	-0.002 (-0,003, -0.001)	0.000
Gender (female)	0.015 (-0.089, 0.120)	0.774	0.002 (-0.103, 0.107)	0.972	0.013 (-0.026, 0.052)	0.509	0.007 (-0.320, 0.045)	0.739
Religion (Muslim)	-0.029 (-0.090, 0.033)	0.360	-0.016 (-0.073, 0.412)	0.589	-0.009 (-0.035, 0.017)	0.503	-0.004 (-0.028, 0.020)	0.751
Education (basic)								
Middle	0.026 (-0.054, 0.106)	0.530	0.023 (-0.048, 0.094)	0.523	0.011 (-0.020, 0.043)	0.479	0.010 (-0.017, 0.037)	0.465
Higher	0.017 (-0.088, 0.122)	0.746	0.014 (-0.082, 0.109)	0.777	0.001 (-0.048, 0.049)	0.984	-0.001 (-0.45, 0.043)	0.959
Labour (manual)	-0.039 (-0.095, 0.017)	0.173	-0.034 (-0.082, 0.015)	0.179	-0.014 (-0.037, 0.010)	0.250	-0.012 (-0.032, 0.008)	0.245
Not working	-0.276 (-0.444, -0.107)	0.001	-0.277 (-0.429, -0.125)	0.000	-0.107 (-0.175, -0.038)	0.002	-0.107 (-0.179, -0.43)	0.001
Married	0.435 (-0.011, 0.098)	0.121	0.047 (-0.002, 0.097)	0.061	0.018 (-0.008, 0.043)	0.178	0.019 (-0.004, 0.042)	0.101
Family pain	-0.197 (-0.333, -0.061)	0.005	-0.212 (-0.342, -0.084)	0.001	-0.077 (-0.138, -0.016)	0.013	-0.078 (-0.134, -0.021)	0.007
Smoker (never)								
Former	-0.018 (-0.102, 0.065)	0.664	-0.042 (-0.126, 0.043)	0.333	-0.007 (-0.059, 0.007)	0.720	-0.018 (-0.056. 0.020)	0.360
Current	-0.085 (-0.206, 0.37)	0.171	-0.083 (-0.196, 0.030)	0.148	-0.036 (-0.023, 0.024)	0.133	-0.036 (-0.081, 0.008)	0.108
Alcohol Drinker (never)								
Former	-0.066 (-0.014, 0.009)	0.083	-0.058 (-0.127, 0.006)	0.075	-0.026 (-0.059, 0.007)	0.119	-0.023 (-0.051, 0.005)	0.108
Current	-0.011 (-0.65, 0.043)	0.694	-0.022 (-0.072, 0.028)	0.389	0.001 (-0.023, 0.024)	0.962	-0.004 (-0,026, 0.017)	0.678
Ever pregnant	-0.073 (-0.167, 0.021)	0.130	-0.065 (-0.161, 0.031)	0.186	-0.032 (-0.066, 0.001)	0.055	-0.029 (-0.063, 0.005)	0.093
Diabetes	0.067 (-0.169, 0.303)	0.579	0.046 (-0.161, 0.253)	0.664	0.032 (-0.62, 0.126)	0.505	0.023 (-0.059, 0.105)	0.588
Hypertension	0.019 (-0.194, 0.233)	0.858	0.007 (-0.179, 0.193)	0.944	0.007 (-0.071, 0.086)	0.856	0.002 (-0.068, 0.072)	0.954
Comorbidities + 1	0.026 (-0.176, 0.228)	0.803	0.028 (-0.146, 0.203)	0.751	0.014 (-0.061, 0.089)	0.707	0.015 (-0.050, 0.081)	0.649
+2	-0.070 (-0.492, 0.351)	0.743	-0.044 (-0.416, 0.327)	0.816	-0.026 (-0.195, 0.144)	0.765	-0.016 (-0.166, 0.135)	0.837
Constant	1.278		1.271		1.11		1.10	
**QoL utility score**	REMS +ve 0.624 (0.563, 0.684)<br>Controls 0.850 (0.823, 0.878)		REMS +ve 0.637 (0.582, 0.693)<br>Controls 0.861 (0.837, 0.885)		REMS +ve 0.845 (0.820, 0.870)<br>Controls 0.940 (0.928, 0.952)		REMS +ve 0.850 (0.827, 0.874)<br>Controls 0.945 (0.934, 0.955)	

[4] Musculoskeletal (MSK)

For peer review only - http://bmjopen.bmj.com/site/about/guidelines.xhtml

Discussion

Our findings reveal a major reduction in HRQOL for people living with musculoskeletal disorders as identified through REMS screening in comparison to those without musculoskeletal in the Hai district of Tanzania. A good quality of life refers to a person's ability to look after themselves, get around their community, participate in their usual activities, and avoid pain and distress. Many musculoskeletal disorders such as rheumatoid arthritis disproportionately affect women(21). Here, we find those presenting with musculoskeletal conditions to be predominantly female, and to have a significant reduction in HRQOL scores, which declines further with age. It is well documented that musculoskeletal disorders incur considerable costs to society through lost productivity (22), and in this study 'not working'(anymore) is associated with a statistically significantly negative impact on HRQOL too.

That they are also more likely to have other conditions has led researchers to study associated patterns of multimorbidity in those with arthritis(23). The reduction of 0.22 utilities in HRQOL found in our study between those screened positive for musculoskeletal disorders and those who did not is of similar magnitude to the reduction in quality of life for people with three or more longstanding conditions living in areas of high socio-economic deprivation in Scotland compared to the general population(24). In that study, Lawson et al observed a similar utility decrement of 0.21. The fact that the drop in utility found due to musculoskeletal disorders is comparable to that of people living with multiple long-term conditions highlights the importance of musculoskeletal health for the quality of life of patients.

Evidence from other countries shows a wide range of utility reductions in those with rheumatoid arthritis. For example, Hoshi et al (25) find studies range from utility scores of 0.49 to 0.75, likely due to differences in patient populations and study designs, with more recent studies having higher HRQOL values, likely reflecting improved management of patients over the last decade. A systematic review of HRQOL scores in rheumatoid arthritis from Asia found those with moderate to high disease activity scores had EQ-5D utilities of 0.53 and 0.47 respectively which were positively associated with age (26). However, utility values significantly differed between the countries in which studies were conducted, including India averaging 0.40, Taiwan 0.67 and Thailand 0.81. They also found EQ-5D utility values significantly differed between study designs, with EQ-5D values for case-control of 0.65 (0.49-0.80), cohort studies 0.60 (0.54-0.66) and cross-sectional studies 0.72 (0.68-0.75)(26). The use of other HRQOL tools to generate utilities may also produce variation. Uhlig et al find found a utility reduction using the Short Form Survey 36 of 0.20 in patients aged 50–60 years with musculoskeletal conditions compared to the general population in Norway (27).

Different methodologies to produce value sets may also account in part for variation in results (28). A limitation of this study is that Tanzania has no value set for utilities. Instead, we used the weights generated in neighbouring Uganda to value health profiles. We have, for this reason, undertaken a sensitivity analysis using the value sets from Ethiopia, another East African country. Although each country employed a combination of slightly different methods, both value sets included the use of time trade-off to generate weights to health profiles where respondents choose between living in a certain impaired health state for a certain length of time, or in full health for a shorter time period (29). In the Ugandan value set, 'pain and discomfort' was the most important dimension with a decrement of 0.798 for the most extreme level. In Ethiopia, 'pain and discomfort' was the second highest contributor to HRQOL reductions, but with a relatively smaller of decrement of 0.406. Musculoskeletal disorders include a range of different conditions and affect people differently, with pain being the most commonly reported symptom, albeit highly subjective and variable. In studies

For peer review only

For peer review only - http://bmjopen.bmj.com/site/about/guidelines.xhtml

exploring HRQOL across multiple chronic diseases, pain has been found to have the largest negative impact on HRQOL (30) with musculoskeletal disorders associated with the largest losses of HRQOL(31). Carreño et al (32) found 'pain or discomfort' to be the highest scoring dimension, with a maximum score in over 12.3% of cases. Our study shows a similar trend with the highest number of extreme scores found in the 'pain or discomfort' and 'mobility' domains equally. Given 'pain or discomfort' is also the most important dimension in the value set used (13), this dimension is driving utility values on both fronts. With such a baseline figure, any future interventions aimed at alleviating symptoms including pain, or indeed any preventive interventions, will likely improve this HRQOL profile.

The dimension 'anxiety and depression' is another area of notable contrast between the two countries' value sets. In Uganda 'anxiety and depression' was found to be the least valued on health. In contrast, in Ethiopia 'anxiety and depression' was rated the most valued. The authors of the Ugandan study note that in Ugandan culture, it is generally considered a weakness for someone to show signs of mental illness or even the need for mental healthcare(13). Indeed, local clinicians in Tanzania have found while collecting HRQOL data in patients that no-one will score high (severely, extremely impacted) for 'anxiety and depression' given the stigma associated with either recognising or talking about such issues publicly[5]. The fact that mental health is traditionally 'taboo' to talk about in some cultures and religions, is likely to affect the scoring for the 'anxiety and depression' dimension in countries such as Tanzania. Whilst the overall population norms in our study approximate the utility ranges for corresponding age groups from a range of other countries (33), it is notable that our controls have a consistently higher utility score across age bands than might otherwise have been expected, especially in the older age groups. In our study, 'anxiety and depression' had the least important scores, and it is likely that utility values are therefore over-estimated in this population (34).

Good practice would dictate that when analysing international studies, country specific value sets should be used to generate utilities valid for that country. In various country comparisons, systematic differences have been found as well as discrepancies when analysing changes from one health state to another health state, not just in terms of magnitude but even in the direction of the change (28). Within these East Africa value sets, generated using similar methods, it is more likely that cultural and contextual reasons are driving a variance in results. In Uganda, the mean for the study sample generating the value set was 0.86 compared with 0.94 in Ethiopia. The authors of the Ethiopian study note evidence of clustering of values at 1 with non-trading of time for milder health states(12). This is evident in the distribution of utilities (see Supplementary File 2). Overall, compared to the Ethiopian value set, the extreme health states had lower values in Uganda. To overcome these limitations and as part of our wider NIHR study, there is ongoing research to develop an African specific measure of health and wellbeing. This measure will be based on the capability approach (35), a multidimensional approach to wellbeing, to understand what aspects of life are important to people in Tanzania using values from people in Tanzania. Arthritis has potential to affect many aspects of the capabilities that are important to people. When completed, this measure can then be used to provide information to decision-makers about which health and care interventions will most benefit people's lives in Tanzania.

Despite these methodological limitations and varying magnitude of results, it is evident that the impact of musculoskeletal conditions on HRQOL is considerable. Decision makers need information on both costs associated with a condition and the health improvements that can be achieved by intervening for those affected by musculoskeletal disorders when planning for cost effective services. Working across different disciplines, and in both clinical and community settings, our wider NIHR

[5] Personal communication

For peer review only - http://bmjopen.bmj.com/site/about/guidelines.xhtml

research project has estimated the prevalence of musculoskeletal disorders, its economic burden and impact on HRQOL[6]. In doing so, we provide an initial evidence-base to justify the need to plan effective and cost-effective interventions for the prevention and management of arthritis and musculoskeletal conditions in Tanzania and wider Sub-Saharan Africa.

Conclusion

This study quantifies the significant HRQOL burden caused by musculoskeletal disorders in Kilimanjaro, and likely wider Tanzania. This is the first powered study of its kind in musculoskeletal disorders in Tanzania (and likely wider SSA) using valid screening tools along with estimates of preference-based quality of life. The results will be used to guide clinical health care delivery, interventions design, service provisions, and health promotion and awareness activities both at institutional level, Kilimanjaro Region and national level. The findings from this study will inform those leading the NCD strategies in Tanzania of the magnitude of the impact of arthritis and related musculoskeletal conditions to plan properly for the management of these disorders, develop bespoke interventions to prevent and reduce the impact of these conditions and to improve quality of life for those who live with and suffer from them.

Funding
This study/project was funded by the UK's NIHR Global Health Research Groups Programme (Award no: 17/63/35). The views expressed are those of the author(s) and not necessarily those of the NIHR or the Department of Health and Social Care.

Data sharing

MD and SK have accessed and checked the underlying data. The data that support the findings of this study are available upon reasonable request.

Competing interests None declared.

Author contributions

Conception and design of the study: BM, EM

Data acquisition: SJK, JH, RW, BM

Data analysis: EG, MD, SJK, PH

Data interpretation: EG, MD, SJK, JH, CK, SS, RW, NY, RK, EM

Drafting of the first version of the manuscript: EG

Critical review of the manuscript: All

All authors read and approved the final manuscript.

Acknowledgements

The NIHR Research Group comprises (in alphabetical order): Sanjura Biswaro, Christopher Bunn, Edith Chikumbu, Jo Coast, Mia Crampin, Manuela Deidda, Eleanor Grieve, Jo Halliday, Viktor Katiti, Clive Kelly, Kajiru Kilonzo, Emma Laurie, Emma McIntosh, Blandina Theophil Mmbaga, Gloria Moshi,

[6] Prevalence and resource use papers in submission/under review.

For peer review only - http://bmjopen.bmj.com/site/about/guidelines.xhtml

Elizabeth Msoka, Febronia Shirima, Stefan Siebert, Jennika Virhia, Richard Walker and Sally Wyke. We are grateful for all the support received for this research, including that from our study participants.

For peer review only

For peer review only - http://bmjopen.bmj.com/site/about/guidelines.xhtml

References

1. Eden S, Renaud F, Flora S, Jean S, Hervé D, Laurent A. The world-wide burden of musculoskeletal diseases: a systematic analysis of the World Health Organization Burden of Diseases Database. Annals of the Rheumatic Diseases. 2019;78(6):844.
2. Arthritis as a potential barrier to physical activity among adults with obesity--United States, 2007 and 2009. MMWR Morb Mortal Wkly Rep. 2011;60(19):614-8.
3. Klinedinst NJ, Resnick B, Yerges-Armstrong LM, Dorsey SG. The Interplay of Genetics, Behavior, and Pain with Depressive Symptoms in the Elderly. Gerontologist. 2015;55 Suppl 1(Suppl 1):S67-77.
4. Briggs AM, Cross MJ, Hoy DG, Sànchez-Riera L, Blyth FM, Woolf AD, et al. Musculoskeletal Health Conditions Represent a Global Threat to Healthy Aging: A Report for the 2015 World Health Organization World Report on Ageing and Health. Gerontologist. 2016;56 Suppl 2:S243-55.
5. Blyth FM, Noguchi N. Chronic musculoskeletal pain and its impact on older people. Best Pract Res Clin Rheumatol. 2017;31(2):160-8.
6. Global, regional, and national incidence, prevalence, and years lived with disability for 328 diseases and injuries for 195 countries, 1990-2016: a systematic analysis for the Global Burden of Disease Study 2016. Lancet. 2017;390(10100):1211-59.
7. Doherty M, Dacre J, Dieppe P, Snaith M. The 'GALS' locomotor screen. Ann Rheum Dis. 1992;51(10):1165-9.
8. Coady D, Walker D, Kay L. Regional Examination of the Musculoskeletal System (REMS): a core set of clinical skills for medical students. Rheumatology (Oxford). 2004;43(5):633-9.
9. United Nations. Designing Household Survey Samples: United Nations; 2008.
10. Kilonzo K, Krauth S, Halliday J, Kelly C, Siebert S, Temu G, et al. Estimating the prevalence, quality of life, economic and societal impact of arthritis in Tanzania: Protocol for a mixed methods study. protocols.io https://dx.doi.org/10.17504/protocols.io.8epv5j8r4l1b/v1; 2023.
11. Feng YS, Kohlmann T, Janssen MF, Buchholz I. Psychometric properties of the EQ-5D-5L: a systematic review of the literature. Qual Life Res. 2021;30(3):647-73.
12. Welie AG, Gebretekle GB, Stolk E, Mukuria C, Krahn MD, Enquoselassie F, et al. Valuing Health State: An EQ-5D-5L Value Set for Ethiopians. Value in Health Regional Issues. 2020;22:7-14.
13. Yang F, Katumba KR, Roudijk B, Yang Z, Revill P, Griffin S, et al. Developing the EQ-5D-5L Value Set for Uganda Using the 'Lite' Protocol. Pharmacoeconomics. 2022;40(3):309-21.
14. Ratcliffe J, Huynh E, Stevens K, Brazier J, Sawyer M, Flynn T. Nothing About Us Without Us? A Comparison of Adolescent and Adult Health-State Values for the Child Health Utility-9D Using Profile Case Best–Worst Scaling. Health Economics. 2016;25(4):486-96.
15. Pearce N. Analysis of matched case-control studies. BMJ. 2016;352:i969.
16. Azur MJ, Stuart EA, Frangakis C, Leaf PJ. Multiple imputation by chained equations: what is it and how does it work? Int J Methods Psychiatr Res. 2011;20(1):40-9.
17. Laurie E, Virhia J, Yongolo N, Biswaro S, McIntosh E, Mmbaga B. Activating Equitable Engagement: from research to policy (and back again): a report on dissemination activities for 'NIHR Global Health Research Group on estimating the prevalence, quality of life, economic and societal impact of arthritis in Tanzania. In: Team NGH, editor. unpublished: https://eprints.gla.ac.uk/298157/; 2023.
18. Yongolo NM, Halliday J, Bunn C, Mtesha B, Kelly C, Krauth SJ, et al. Estimating the prevalence and predictors of musculoskeletal disorders in Tanzania: a cross-sectional pilot study. Pan Afr Med J. 2024;47:36.
19. Msoka EF, Bunn C, Msoka P, Yongolo NM, Laurie E, Wyke S, et al. Rapid ethnographic appraisal of community concepts of and responses to joint pain in Kilimanjaro, Tanzania. BMJ Glob Health. 2024;9(1).
20. Devlin N, Finch AP, Parkin D. Guidance to Users of EQ-5D-5L Value Sets. In: Devlin N, Roudijk B, Ludwig K, editors. Value Sets for EQ-5D-5L: A Compendium, Comparative Review & User Guide. Cham: Springer International Publishing; 2022. p. 213-33.

For peer review only - http://bmjopen.bmj.com/site/about/guidelines.xhtml

21. Angum F, Khan T, Kaler J, Siddiqui L, Hussain A. The Prevalence of Autoimmune Disorders in Women: A Narrative Review. Cureus. 2020;12(5):e8094.
22. Hsieh P-H, Wu O, Geue C, McIntosh E, McInnes IB, Siebert S. Economic burden of rheumatoid arthritis: a systematic review of literature in biologic era. Annals of the Rheumatic Diseases. 2020;79(6):771.
23. McLoone P, Jani BD, Siebert S, Morton FR, Canning J, Macdonald S, et al. Classification of long-term condition patterns in rheumatoid arthritis and associations with adverse health events: a UK Biobank cohort study. J Multimorb Comorb. 2023;13:26335565221148616.
24. Lawson KD, Mercer SW, Wyke S, Grieve E, Guthrie B, Watt GC, et al. Double trouble: the impact of multimorbidity and deprivation on preference-weighted health related quality of life a cross sectional analysis of the Scottish Health Survey. Int J Equity Health. 2013;12:67.
25. Hoshi D, Tanaka E, Igarashi A, Inoue E, Kobayashi A, Sugimoto N, et al. Profiles of EQ-5D utility scores in the daily practice of Japanese patients with rheumatoid arthritis; Analysis of the IORRA database. Mod Rheumatol. 2016;26(1):40-5.
26. Haridoss M, Bagepally BS, Natarajan M. Health-related quality of life in rheumatoid arthritis: Systematic review and meta-analysis of EuroQoL (EQ-5D) utility scores from Asia. Int J Rheum Dis. 2021;24(3):314-26.
27. Uhlig T, Loge JH, Kristiansen IS, Kvien TK. Quantification of reduced health-related quality of life in patients with rheumatoid arthritis compared to the general population. J Rheumatol. 2007;34(6):1241-7.
28. Gerlinger C, Bamber L, Leverkus F, Schwenke C, Haberland C, Schmidt G, et al. Comparing the EQ-5D-5L utility index based on value sets of different countries: impact on the interpretation of clinical study results. BMC Research Notes. 2019;12(1):18.
29. Lugnér AK, Krabbe PFM. An overview of the time trade-off method: concept, foundation, and the evaluation of distorting factors in putting a value on health. Expert review of pharmacoeconomics & outcomes research. 2020;20(4):331-42.
30. Wu M, Brazier JE, Kearns B, Relton C, Smith C, Cooper CL. Examining the impact of 11 long-standing health conditions on health-related quality of life using the EQ-5D in a general population sample. The European journal of health economics : HEPAC : health economics in prevention and care. 2015;16(2):141-51.
31. Saarni SI, Härkänen T, Sintonen H, Suvisaari J, Koskinen S, Aromaa A, et al. The impact of 29 chronic conditions on health-related quality of life: a general population survey in Finland using 15D and EQ-5D. Qual Life Res. 2006;15(8):1403-14.
32. Carreño A, Fernández I, Badia X, Varela C, Roset M. Using HAQ-DI to estimate HUI-3 and EQ-5D utility values for patients with rheumatoid arthritis in Spain. Value Health. 2011;14(1):192-200.
33. Janssen MF, Szende A, Cabases J, Ramos-Goñi JM, Vilagut G, König HH. Population norms for the EQ-5D-3L: a cross-country analysis of population surveys for 20 countries. Eur J Health Econ. 2019;20(2):205-16.
34. Lugata S, Elinisa M, Doshi B, Kashuta RA, Hango S, Mallosa WJ, et al. Symptoms and predictors of depression among university students in the Kilimanjaro region of Tanzania: a cross-sectional study. Journal of Mental Health. 2021;30(2):255-62.
35. Coast J, Kinghorn P, Mitchell P. The development of capability measures in health economics: opportunities, challenges and progress. Patient. 2015;8(2):119-26.

For peer review only - http://bmjopen.bmj.com/site/about/guidelines.xhtml

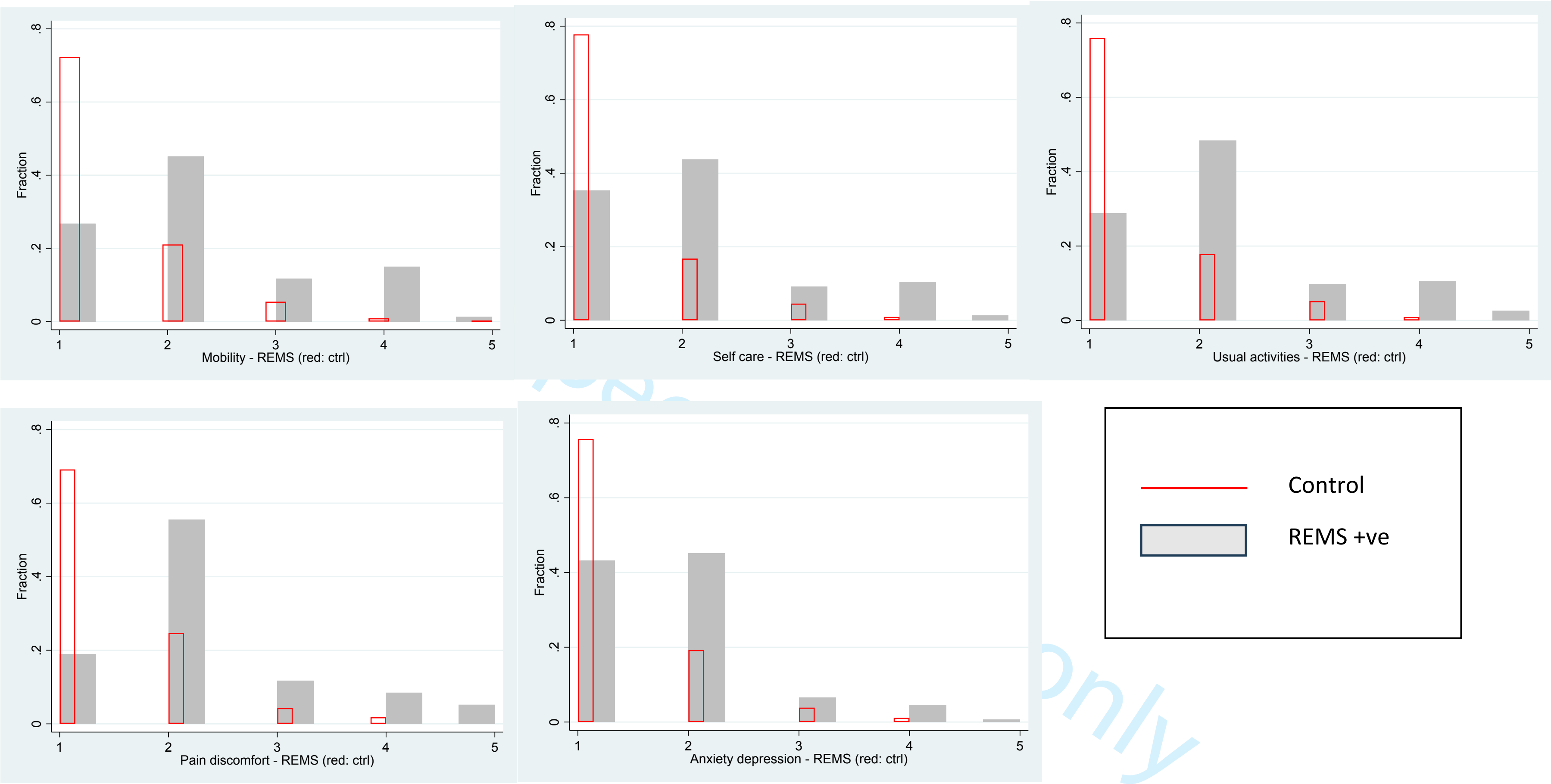

Figure 1: Score distributions for those identified positive as having a musculoskeletal disorder compared with matched controls for each HRQOL dimension (Ugandan tariff)

Supplementary 1: Model fit tests

GLMDIAGL: This program runs the modified Park test, Pearson correlation test, Pregibon link test, and modified Hosmer and Lemeshow test, for a set of power links that range between -2 and 2 in intervals of 0.1. It records the results of these tests as well as the log likelihood, AIC, BIC statistics from each power model. Saved statistics are for the model that uses the family specified by the modified Park test. [glmdiagl "glm QoLUg i.rems_ctrl age i.sex i.religion i.edusimple i.labour i.marrydummy i.familypain i.intsmoke i.intdrink i.everpregnant i.diabetest3 i.hypertensiont3 i.MM, vce(cluster clustervar)"]

```
                 _________________ Summary Score Recommendations_________________
                   3-(sum p)      sum abs|log(p)|   3-(sum p^.5)       sum(1-p)^2
Ranking          Link    Family    Link    Family   Link    Family    Link    Family

Preferred        1.00       0     -2.00       0     1.00       0      1.00       0
2nd best         0.90       0     -1.90       0     0.90       0      0.90       0
3rd best         0.80       0     -1.80       0     0.80       0      0.80       0

See r(linkmat1), r(linkmat2), and r(familymat) for more detailed results

Family Coding (for r(familymat)

0 = gaussian; 1 = poisson; 2 = gamma; 3 =igaussian; 4 = Preferred family switches
''Conflicting families'': 40 = gaussian; 41 = poisson; 42 = gamma; 43 = igaussian
''Initial values infeasible'': 70 = gaussian; 71 = poisson; 72 = gamma; 73 = igaussian;
5 = None
```

Source: https://drhenryglick.com/identifying-better-links-and-families/

```
 FITTED MODEL:  Link = Power  1; Family =  Gaussian

 Results, Modified Park Test (for Family)

    Coefficient:   -2.60235

    Family, Chi2, and p-value in descending order of likelihood

      Family                           Chi2         P-value

      Gaussian NLLS:                86.6945          0.0000
      Poisson:                     166.1237          0.0000
      Gamma:                       271.1560          0.0000
      Inverse Gaussian or Wald:    401.7912          0.0000

L
 Results of tests of GLM Power  1 link

      Pearson Correlation Test:                      1.0000
      Pregibon Link Test:                            0.0006
      Modified Hosmer and Lemeshow:                  0.0000
      SS: sqr1_p                                     1.9988
      SS: sqrtp                                      1.9755
      SS: logp                                      32.7470
      SS: sump                                       1.9994
      R-square                                       0.3478
      Log likelihood                               -52.9043
      AIC                                            0.2865
      BIC                                        -2880.1483
      Deviance                                      35.4903


. **Gaussian family given identity link
```

Supplementary 2: Distributions of Utilities

Distribution of utilities using Ugandan and Ethiopian tariffs: population

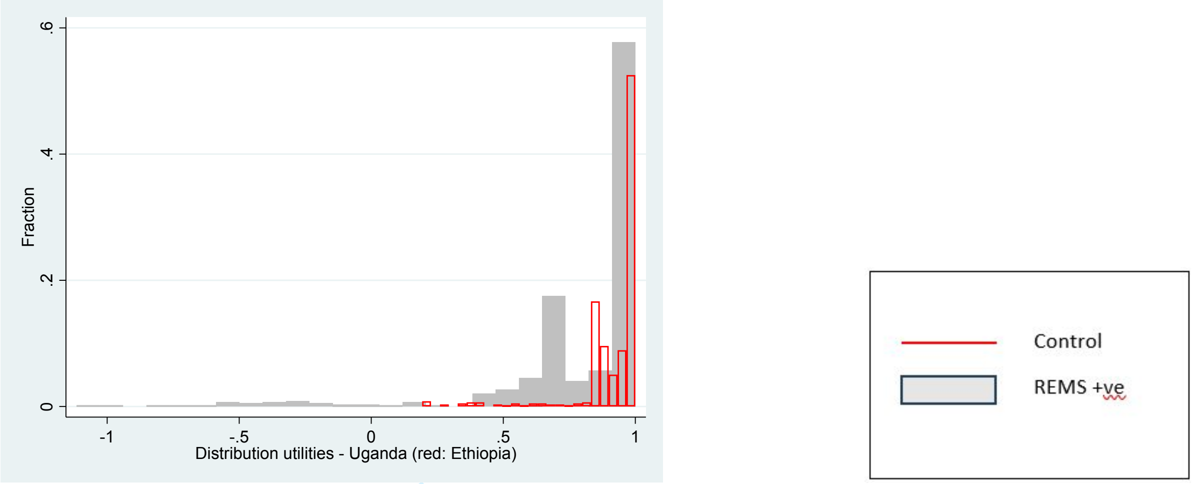

Distribution of utilities using Ugandan and Ethiopian tariffs: REMS positive

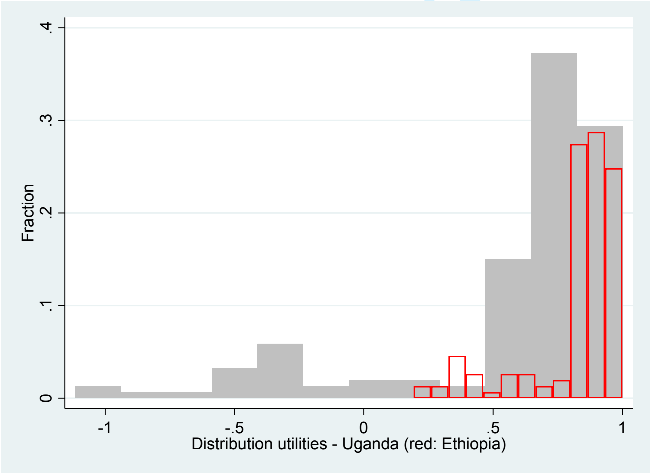

Distribution of utilities using Ugandan and Ethiopian tariffs: Controls

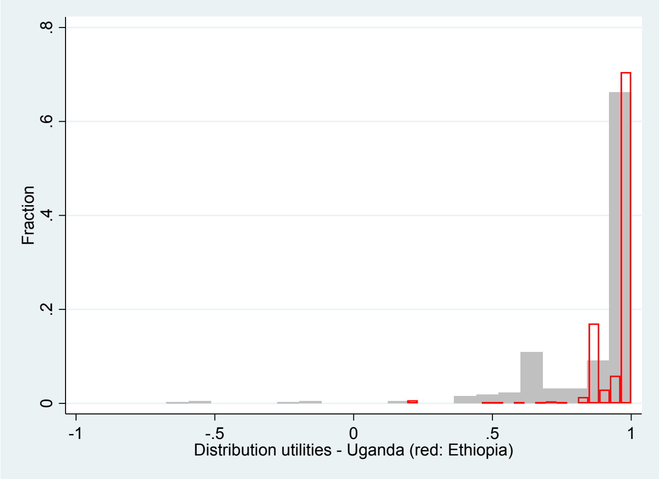

For peer review only

How do Musculoskeletal Disorders impact on Quality of Life in Tanzania? Results from a Community-Based Survey

Journal:	*BMJ Open*
Manuscript ID	bmjopen-2024-092877.R1
Article Type:	Original research
Date Submitted by the Author:	09-Oct-2025
Complete List of Authors:	Grieve, Eleanor; University of Glasgow College of Medical Veterinary and Life Sciences, School of Health and Wellbeing, Health Economics and Health Technology Assessment<br>Deidda, Manuela; University of Glasgow College of Medical Veterinary and Life Sciences, School of Health and Wellbeing, Health Economics and Health Technology Assessment<br>Krauth, Stefanie; University of Glasgow, School of Health and Wellbeing, College of Medical, Veterinary and Life Sciences; Canterbury Christ Church College, School of Allied and Public Health Professions<br>Biswaro, Sanjura Mandela; Kilimanjaro Christian Medical Centre; Kilimanjaro Christian Medical College<br>Halliday, J; University of Glasgow College of Medical Veterinary and Life Sciences, School of Biodiversity, One Health and Veterinary Medicine<br>Hsieh, Ping-Hsuan; National Defense Medical Center<br>Kelly, Clive; James Cook University Hospital; Newcastle University<br>Kilonzo, Kajiru; Kilimanjaro Clinical Research Institute, Kilimanjaro Christian Medical Center; Kilimanjaro Christian Medical University College<br>Kiula, Kiula P.; Kilimanjaro Clinical Research Institute; Institute of Rural Development Planning, Rural Development and Regional Planning<br>Kolimba, Rose; Kilimanjaro Clinical Research Institute, Kilimanjaro Christian Medical Center<br>Msoka, Elizabeth ; Kilimanjaro Christian Medical University College; Kilimanjaro Christian Medical University College<br>Siebert, Stefan; University of Glasgow College of Medical Veterinary and Life Sciences, School of Infection and Immunity<br>Walker, Richard William; Newcastle University<br>Yongolo, Nateiya; Kilimanjaro Christian Medical Centre, Clinical Research; Liverpool School of Tropical Medicine Department of Clinical Sciences,<br>Mmbaga, Blandina; Kilimanjaro Christian Medical University College, ; Kilimanjaro Clinical Researxh Institute<br>McIntosh, Emma; University of Glasgow College of Medical Veterinary and Life Sciences, School of Health and Wellbeing, Health Economics and Health Technology Assessment<br>NIHR Global Health Group, On behalf of; NIHR
**Primary Subject Heading**:	Health economics
Secondary Subject Heading:	Rheumatology, Global health, Health policy, Health services research, Research methods

Keywords:	Musculoskeletal disorders < ORTHOPAEDIC & TRAUMA SURGERY, HEALTH ECONOMICS, Quality of Life, Cross-Sectional Studies

SCHOLARONE™
Manuscripts

I, the Submitting Author has the right to grant and does grant on behalf of all authors of the Work (as defined in the below author licence), an exclusive licence and/or a non-exclusive licence for contributions from authors who are: i) UK Crown employees; ii) where BMJ has agreed a CC-BY licence shall apply, and/or iii) in accordance with the terms applicable for US Federal Government officers or employees acting as part of their official duties; on a worldwide, perpetual, irrevocable, royalty-free basis to BMJ Publishing Group Ltd ("BMJ") its licensees and where the relevant Journal is co-owned by BMJ to the co-owners of the Journal, to publish the Work in this journal and any other BMJ products and to exploit all rights, as set out in our licence.

The Submitting Author accepts and understands that any supply made under these terms is made by BMJ to the Submitting Author unless you are acting as an employee on behalf of your employer or a postgraduate student of an affiliated institution which is paying any applicable article publishing charge ("APC") for Open Access articles. Where the Submitting Author wishes to make the Work available on an Open Access basis (and intends to pay the relevant APC), the terms of reuse of such Open Access shall be governed by a Creative Commons licence – details of these licences and which Creative Commons licence will apply to this Work are set out in our licence referred to above.

Other than as permitted in any relevant BMJ Author's Self Archiving Policies, I confirm this Work has not been accepted for publication elsewhere, is not being considered for publication elsewhere and does not duplicate material already published. I confirm all authors consent to publication of this Work and authorise the granting of this licence.

For peer review only

How do Musculoskeletal Disorders impact on Quality of Life in Tanzania? Results from a Community-Based Survey

Authors: Eleanor Grieve[1,◊], Manuela Deidda[1], Stefanie Krauth[1,2], Sanjura Mandela Biswaro[3], J. E. B. Halliday[4], Ping-Hsuan Hsieh[5], Clive Kelly[6,7], Kajiru Kilonzo[8,9], Kiula P Kiula[8], Rose Kolimba[8], Elizabeth F. Msoka[,8,9], Stefan Siebert[10], Richard William Walker[7], Nateiya Mmeta Yongolo[3,11], Blandina Mmbaga[,8,9], Emma McIntosh[1] and the NIHR Global Health Group[12]

[1] School of Health and Wellbeing, College of Medical, Veterinary and Life Sciences, University of Glasgow, Clarice Pears Building, Glasgow, G12 8TB, Scotland, UK
[2] Canterbury Christ Church College, School of Allied and Public Health Professions, Canterbury, England, UK
[3] Kilimanjaro Christian Medical Centre, Clinical Research, Moshi, Tanzania
[4] School of Biodiversity, One Health and Veterinary Medicine, College of Medical, Veterinary and Life Sciences, University of Glasgow, G12 8QQ, Glasgow, UK
[5] School of Pharmacy, National Defense Medical Center, Taipei City, Taiwan
[6] James Cook University Hospital, Middlesbrough, Marton Rd, Middlesbrough TS4 3BW, England
[7] Newcastle University, Claremont Road, Newcastle upon Tyne NE1 7RU, England
[8] Kilimanjaro Clinical Research Institute, Moshi, United Republic of Tanzania
[9] Kilimanjaro Christian Medical University College, Moshi, United Republic of Tanzania
[10] School of Infection and Immunity, College of Medical, Veterinary and Life Sciences, University of Glasgow, Sir Graham Davies Building, 120 University Place, Glasgow G12 8QQ, Scotland
[11] Liverpool School of Tropical Medicine Department of Clinical Sciences, Liverpool, UK L3 5QA
[12] NIHR Global Health Research Group on Arthritis in Tanzania: nihr-ghrg-arthritis@glasgow.ac.uk

[◊] Corresponding author: Eleanor Grieve, School of Health and Wellbeing, University of Glasgow, Clarice Pears Building, Glasgow, G12 8RZ, Scotland
E-mail: Eleanor.Grieve@glasgow.ac.uk
Access to the data can be requested through the corresponding author

Keywords: Arthritis, joint disorders, musculoskeletal disorders, Tanzania, cross-sectional, health related quality of life, EQ5D

Word count: 3790

For peer review only

Article Summary

Abstract

Objectives
There are little available data on the prevalence, economic and quality of life impacts of musculoskeletal disorders in Sub-Saharan Africa (SSA). This lack of evidence is wholly disproportionate to the significant disability burden of musculoskeletal disorders as reported in high-income countries. Our research aimed to undertake an adequately powered study to identify, measure and value the health impact of musculoskeletal conditions in the Kilimanjaro region, Tanzania.

Design
A community-based cross-sectional survey was undertaken between January 2021 and September 2021. A two-stage cluster sampling with replacement and probability proportional to size was used to select a representative sample of the population.

Setting
The survey was conducted in 15 villages in the Hai District, Kilimanjaro region, Tanzania.

Participants
Economic and health-related quality of life (HRQOL) questionnaires were administered to a sample of residents (aged over 5 years old) in selected household (N = 1050). There was a total of 594 respondents, of whom 153 had a confirmed musculoskeletal disorder and 441 matched controls. Almost three quarters of those identified as having a musculoskeletal disorder were female and had an average age of 66 years.

Primary and secondary outcome measures
Questions on health care resource use, expenditure and quality of life were administered to all participants, with additional more detailed economic and quality of life questions administered to those who screened positive, indicating probable arthritis.

Results
There is a statistically significant reduction in HRQOL, on average 25% from a utility score of 0.862 (0.837, 0.886) to 0.636 (0.580, 0.692) for those identified as having a musculoskeletal disorder compared to those without. The attributes 'pain' and 'discomfort' were the major contributors to this reduction in HRQOL.

Conclusions
This research has revealed a significant impact of musculoskeletal conditions on HRQOL in the Hai district in Tanzania. The evidence will be used to guide clinical health practices, interventions design, service provisions, and health promotion and awareness activities at institutional, regional and national levels.

Strengths and limitations of this study
- This is the first powered study of its kind in musculoskeletal disorders in Tanzania (and likely wider SSA) using valid screening tools along with estimates of preference-based quality of life.
- A two-stage sampling strategy was used to identify a representative sample of residents in the Hai district.
- An economic questionnaire, tailored to the Tanzania context, captured quality of life measures and healthcare resource use was administered to those with likely musculoskeletal (MSK) disorders and selected controls..

For peer review only - http://bmjopen.bmj.com/site/about/guidelines.xhtml

- A limitation of this study is that Tanzania has no value set for utilities. Instead, we used the weights generated in neighbouring Uganda to value health profiles and undertook a sensitivity analysis using the value sets from Ethiopia.

Background

Musculoskeletal disorders are ranked as the second most common cause of disability worldwide[1]. These conditions cause not only clinical but economic, societal, and quality of life impacts on people's day-to-day lives [2]. Furthermore, musculoskeletal conditions often predispose and directly contribute to other comorbidities and non-communicable diseases (NCDs) [3]. Epidemiological studies confirm the strong relationship between painful musculoskeletal conditions, lack of physical activity and resulting functional decline with frailty, loss of wellbeing and loss of independence and depressive symptoms [4 5]. Musculoskeletal health is critical for people's mobility and dexterity, their ability to work and actively participate in all aspects of life and to maintain economic, social and functional independence across their life course [2]. Musculoskeletal conditions have suffered from a distinct lack of attention from the medical profession, politicians, and the public because they are not usually life threatening and many are considered to be a natural part of aging, despite being the most prevalent causes of disability, absence from occupational activities and early retirement impacting both the individual and also wider society [6].

However, little evidence exists on the prevalence, patterns and impacts of such conditions among the general population in Sub-Saharan Africa (SSA) [2]. Further, existing strategic plans to reduce NCDs in this setting often overlook the impact of musculoskeletal disorders [7]. When prioritising healthcare research and investment, the burden of musculoskeletal disorders has been underestimated and even ignored, mainly due to their chronic nature and perceived low fatality rate [8]. Whilst the importance and severity of the emerging NCD burden is well recognised with strategic national action plans in place for the prevention and control of NCDs, the focus is dominated by conditions such as cardiovascular diseases, cancer and diabetes [9]. Little attention has been paid to musculoskeletal conditions. This is out of proportion to the impact that musculoskeletal conditions have on disability, with musculoskeletal being one of the major causes of non-traumatic disability within the global burden of disease [10]. Arthritis (joint diseases)are among the commonest musculoskeletal disorders and are a leading cause of physical disability and severe long-term pain [11]. Measuring the full impact of these disorders is urgently needed to inform policy.

Tanzania has made progressive commitments towards Universal Health Coverage (UHC) with the scale up of health insurance to be rolled out in 2023 and has launched a National NCDs strategic plan. However, plans often prioritise other NCDs conditions such as diabetes, hypertension and sickle cell disease whilst leaving musculoskeletal conditions behind. The 'NIHR Global Health Research Group on estimating the prevalence, quality of life, economic and societal impact of arthritis in Tanzania: a mixed methods study at University of Glasgow was established with the aim of characterising and estimating the various burdens of arthritis in Tanzania. This wide-ranging study aimed to establish the prevalence of arthritis and joint problems and, to better understand their impact - clinically, economically and socially - in Tanzania. Here, we present the findings related to the impact on health-related quality of life (HRQOL) for those with musculoskeletal conditions and arthritis in Tanzania.

Methods

A community-based cross-sectional survey conducted in 15 villages in the Hai District, Kilimanjaro was undertaken between January 2021 and September 2021 working in partnership with the Kilimanjaro

For peer review only - http://bmjopen.bmj.com/site/about/guidelines.xhtml

Clinical Research Institute (KCRI). Clinical screening tools, including the Gait Arms Legs Spine (GALS) [12] and Regional Examination of the Musculoskeletal system (REMS) [13], were used to screen people for musculoskeletal conditions through a tiered approach. A two-stage cluster sampling with replacement and probability proportional to size was used to select a representative sample of the population[14]. We administered a battery of instruments including economic (i.e. usage of healthcare resources; productivity impact etc.) and HRQOL questions to a sample of residents (aged over 5 years old) in selected household (N = 1050 households). Questions on health care resource use, expenditure and quality of life were administered to all participants, with additional more detailed (long version) economic and quality of life questions administered to those who screened positive on REMS (only GALS positive individuals were assessed with REMS), indicating probable arthritis.

Each person was assessed at their home by two interviewers using the standardised study protocol [15]. The interview commenced with a series of questions prior to the performance of first GALS, then REMS (if GALS was positive). If REMS was positive, then further investigations including blood tests and X rays were subsequently arranged as indicated. Regarding co-morbidities, we asked whether the person had any previous illnesses or required previous hospitalisations. We asked about any medication they took. We also asked specifically about other common conditions including NCDs such as high blood pressure, heart disease, breathlessness, lung disease, urinary issues, kidney disease, as well as about infections such as malaria, tuberculosis and HIV.

In order to establish a reference population for these measures, this group were matched on age (+/- 1 year for minors and +/-3 years for adults) and gender, aiming for 2 controls per REMS positive case from the population of GALS negative participants. In cases where no age match could be identified within this range, e.g. for very old participants, larger age-differences were accepted rather than not recruiting a control. Hence responses from participants screening positive for musculoskeletal conditions were able to be directly compared to a matched control group in a bid to establish the relative magnitude of quality of life impact of the musculoskeletal disorders. Full details are provided in the study protocol [15].

Quality of life tools

The Swahili version of the preference-based tool EuroQol (EQ-5D-5L) was used for those 18 years and over[16]. The EQ-5D is a generic questionnaire relating to mobility, self-care, usual activity, pain and discomfort, and anxiety and depression. The EQ-5D health state is converted into a utility score (typically between 0-1 where 0 represents death and 1 represents full health) using a country-specific scoring algorithm or value set. The value attached to an EQ-5D profile according to a set of weights reflects on average, people's preferences about how good or bad the state is. As Tanzania has no set of weights for the EQ-5D dimensions, the Ugandan tariff was used as the base case and the Ethiopian tariff as a sensitivity analysis[17 18].The Child Health Utility (CHU9D) instrument was used for children and adolescents under 18 years [19].

Model

A generalised linear model (GLM) model with appropriate family/link was fitted to estimate differences in utility scores (dependent variable) between those presenting as REMS positive i.e. with probable musculoskeletal disorder and controls. The modified Park test was conducted to choose the best distribution while a suite of tests was run to guide the best choice of link. See Supplementary File 1 for full details. The primary explanatory variable categorised individuals into MSK condition (REMS+) or control groups. The regressions were also adjusted by demographics (age and gender), socioeconomic (education, occupation and marital status) and clinical/lifestyle (family members who

For peer review only - http://bmjopen.bmj.com/site/about/guidelines.xhtml

had experienced joint pain, smoking and drinking habits, diagnosis of diabetes) variables. For education, participants were asked "What is the highest level of school that you completed: primary/middle/higher?" Lifestyle issues (drinking, smoking habits) were asked if current, former or never". Explanatory variables in the final model included age, occupation, marital status, gender, , education, co-morbidities and behavioural risk factors. Although a matched control group was established, we still controlled for sex and age in our case control model to insure against imperfect matching [20]. Standard errors were adjusted for clustering at household and village level. Multiple imputation procedures using chained equations and all covariates were used to impute missing data separately for each group (MSK/controls), creating 40 imputed datasets[21 22]. Both complete case and multiple imputation analyses were undertaken. All analyses were performed using Stata version 17.

Patient and public involvement

The community were involved throughout this research, from design to dissemination. Please see report which details the dissemination outputs, stakeholder engagement activities and their underlying principles conducted by this study [23].

Ethical considerations

The study was conducted in accordance with national and international ethical guidelines and principles and received clearance from the College of Medical, Veterinary & Life Sciences Ethics Committee at the University of Glasgow (Ref. number: 200180100), the Kilimanjaro Christian Medical University College Local Ethical Review Committee (KCMC/P.I/Vol.XI), and the National Institute for Medical Research in Tanzania (NIMR/HQ.R.8a/Vol.IX/3038). Written informed consent was obtained from all adult participants and guardians of participants aged <18 years after detailed information about the study was provided and time allowed for any questions. In addition to guardian consent, assent was obtained from participants aged 12-17 years. Consent documents were all provided in Swahili. In case of illiteracy or other language requirements, a witness and/or translator could be chosen by the participants to provide verbal translation during consenting and survey delivery. All data collection instruments were translated to Swahili and adapted to the local context[24 25].

Results

There was a total of 594 respondents, of whom 153 had a confirmed musculoskeletal disorder and 441 matched controls. Table 1 provides a descriptive analysis comparing REMS positive individuals and matched controls, in terms of demographic, clinical and socio-economic characteristics. Almost three quarters of those identified as REMS positive were female and had an average age of 66 years. They were twice as likely to not be working than those without a musculoskeletal disorder (12% compared with 6%). Comorbidity was higher among REMS+ patients; they were more likely to have other conditions, in particular, hypertension (24% compared with 14%). Quality of life utility scores by age and count of co-morbidities showed a clear distinction between those with a musculoskeletal disorder, compared to those without a musculoskeletal disorder for all age groups.

In terms of missing data, although only one person did not answer the EQ-5D questionnaire, a 18% rate of missingness in the covariates (especially history of family pain) required the use a of a multiple imputation approach. Two children were positive with GALS, one refused to be examined further with REMS, and the other was REMS positive.

For peer review only - http://bmjopen.bmj.com/site/about/guidelines.xhtml

Table 1: Descriptive summary of characteristics for all participants, and by musculoskeletal /arthritis positive and matched controls.

Categories	All (n = 594)	REMS+ (n = 153)	Controls (n = 441)
Background characteristics			
**Age (mean)**	62.8	66.4	61.5
**Gender (female)**	70%	74%	68%
**Occupation (farmer)**	78%	78%	78%
**Unemployed or retired**	7.24%	11.8%	5.67%
**Religion**			
Christian	82%	79%	84%
Muslim	18%	21%	16%
**Education**			
Basic	87%	91%	86%
Middle	9%	8%	10%
Higher	4%	1%	4%
**Married**	58%	49%	61%
**Smoker**			
Current	7%	5%	8%
Former	9%	10%	9%
Never	84%	85%	83%
**Alcohol Drinker**			
Current	31%	32%	31%
Former	23%	21%	24%
Never	46%	47%	45%
**History of family pain**	9%	25%	3%
Other conditions			
+1 any other condition (n)	18% (96)	26% (39)	15% (57)
+2 any other conditions (n)	3% (18)	3% (5)	3% (13)
Hypertension (n)	17% (89)	24% (37)	14% (52)
Diabetes (n)	5% (29)	5% (8)	6% (21)
Tuberculosis (n)	2% (8)	1% (2)	2% (6)
Quality of life (Utility weight (0-1) [26]			
**All**	0.80 (594)	0.57 (153)	0.88 (441)
**By age:**			
< 30 years (n)	0.94 (15)	0.61 (2)	0.99 (13)
30 – 40 years (n)	0.91 (14)	1.0 (2)	0.90 (12)
40-50 years (n)	0.93 (56)	0.84 (15)	0.96 (41)
50 – 60 years (n)	0.87 (136)	0.73 (29)	0.91 (107)
60 – 70 years (n)	0.83 (191)	0.64 (43)	0.89 (148)
> 70 years (n)	0.67 (182)	0.37 (62)	0.82 (120)
**By comorbidity:**			
+1 condition (n)	0.75 (96)	0.62 (39)	0.85 (57)
+2 conditions (n)	0.68 (18)	0.32 (5)	0.82 (13)

For peer review only - http://bmjopen.bmj.com/site/about/guidelines.xhtml

Table 2 provides the results for both the complete case and multiple imputed datasets using the Ugandan and Ethiopian tariffs. Using the chosen family of Gaussian non-linear least squares with identity link and taking the Ugandan results as the base case, there is a statistically significant reduction in EQ-5D HRQOL, on average 25% from a utility score of 0.86 to 0.64, for those identified as having a musculoskeletal disorder compared to those without. Age, history of family pain and no longer working are associated with a statistically significant reduction in quality of life in both the complete case and multiple imputed analyses. Gender was not a significant factor. Whilst women are more likely to have a musculoskeletal disorder, there was no significant difference between men and women in terms of reduced HRQOL. Comorbidities do not have a statistically significant impact on HRQOL. This is likely due to the relatively low number of co-morbidities in our data.

Using the Ethiopian value set for the purposes of sensitivity analyses, there is again a statistically significant reduction in quality of life, on average of around 10% from a utility score of 0.95 to 0.85, for those identified as REMS positive compared with those who are not. Absolute utility values across both groups are higher using the Ethiopian value set as compared with those generated by the Ugandan value set. Age, history of family pain and no longer working are similarly associated with a statistically significant reduction in quality of life in both the complete case and multiple imputed analyses.

All five dimensions of the EQ-5D - mobility, self-care, usual activity, pain and discomfort, and anxiety and depression - were scored lower by those with a positive diagnosis. Notably, 'pain and discomfort' was a major contributor to this reduction in quality of life. The distribution of each HRQOL dimension (for the Ugandan tariff only) is shown in Figure 1. The controls (in red) show a similar distribution across each domain skewed towards the scores indicating 'none' or 'mild' problems, in contrast the pattern for those with musculoskeletal indicates greater numbers scoring moderate and severe problems in each domain.

Insert Figure 1: Score distributions for those identified positive as having a musculoskeletal disorder compared with matched controls for each HRQOL dimension (Ugandan tariff)

Legend

For peer review only

For peer review only - http://bmjopen.bmj.com/site/about/guidelines.xhtml

Table 2: EQ-5D scores and associated decrements for those identified positive as having a musculoskeletal disorder compared with matched controls – complete case and multiple imputation using Ugandan (base case) and Ethiopian (sensitivity analysis) tariffs.

	Ugandan tariff				Ethiopian tariff			
**Categories**	**Quality of life decrements (Complete case) N = 488**		**Quality of life decrements (Multiple imputation) N = 594**		**Quality of life decrements (Complete case) N = 488**		**Quality of life decrements (Multiple imputation) N = 594**	
	**QoL (95% CI)**	**p-value**	**QoL (95% CI)**	**p-value**	**QoL (95% CI)**	**p-value**	**QoL (95% CI)**	**p-value**
MSK[1] (REMS+)	-0.227 (-0.297, -0.158)	0.000	-0.225 (-0.288, -0.162)	0.000	-0.095 (-0.124, -0.065)	0.000	-0.094 (-0.120, -0.068)	0.000
Age	-0.005 (-0.007, -0.003)	0.000	-0.005 (-0.007, -0.003)	0.000	-0.002 (-0.003, -0.001)	0.000	-0.002 (-0,003, -0.001)	0.000
Gender (female)	-0.056 (-0.114, 0.002)	0.057	-0.063 (-0.116, -0.009)	0.021	-0.019 (-0.043, 0.004)	0.112	-0.022 (-0.045, -0.000)	0.050
Education (basic)								
Middle	0.024 (-0.054, 0.101)	0.550	0.020 (-0.050, 0.090)	0.580	0.010 (-0.020, 0.040)	0.511	0.009 (-0.018, 0.036)	0.507
Higher	0.017 (-0.088, 0.123)	0.744	0.012 (-0.083, 0.107)	0.807	-0.000 (-0.049, 0.049)	0.993	-0.002 (-0.047, 0.042)	0.919
Labour (manual)	-0.041 (-0.097, 0.014)	0.140	-0.037 (-0.086, 0.012)	0.135	-0.015 (-0.037, 0.008)	0.202	-0.013 (-0.033, 0.007)	0.193
Not working	-0.282 (-0.449, -0.114)	0.001	-0.281 (-0.432, -0.129)	0.000	-0.108 (-0.177, -0.039)	0.002	-0.107 (-0.171, -0.044)	0.001
Married	0.041 (-0.014, 0.096)	0.121	0.045 (-0.004, 0.094)	0.074	0.016 (-0.009, 0.042)	0.206	0.018 (-0.005, 0.040)	0.123
Family pain	-0.197 (-0.335, -0.059)	0.005	-0.208 (-0.339, -0.077)	0.002	-0.076 (-0.137, -0.016)	0.014	-0.079 (-0.136, -0.023)	0.006
Smoker (never)								
Former	-0.021 (-0.103, 0.062)	0.626	-0.043 (-0.125, 0.039)	0.301	-0.007 (-0.046, 0.031)	0.701	-0.018 (-0.056. 0.021)	0.365
Current	-0.088 (-0.212, 0.035)	0.171	-0.086 (-0.200, 0.029)	0.142	-0.037 (-0.085, 0.011)	0.127	-0.036 (-0.081, 0.008)	0.117
Alcohol Drinker (never)								
Former	-0.066 (-0.141, 0.008)	0.082	-0.059 (-0.124, 0.006)	0.075	-0.027 (-0.059, 0.005)	0.094	-0.025 (-0.052, 0.003)	0.078
Current	-0.009 (-0.059, 0.041)	0.717	-0.023 (-0.069, 0.023)	0.328	0.000 (-0.021, 0.021)	0.980	-0.006 (-0,026, 0.014)	0.530
Diabetes	0.034 (-0.074, 0.143)	0.537	0.032 (-0.165, 0.128)	0.521	0.021 (-0.023, 0.64)	0.357	0.017 (-0.023, 0.056)	0.411
Hypertension	0.028 (-0.054, 0.110)	0.503	0.021 (-0.054, 0.096)	0.590	0.014 (-0.022, 0.050)	0.443	0.010 (-0.022, 0.043)	0.540
Constant	1.282		1.281		1.109		1.110	
**QoL utility score**	REMS +ve 0.623 (0.562, 0.684)<br>Controls 0.850 (0.823, 0.878)		REMS +ve 0.636 (0.580, 0.692)<br>Controls 0.862 (0.837, 0.886)		REMS +ve 0.845 (0.820, 0.870)<br>Controls 0.940 (0.928, 0.952)		REMS +ve 0.850 (0.827, 0.874)<br>Controls 0.945 (0.934, 0.955)	

Musculoskeletal (MSK)

For peer review only - http://bmjopen.bmj.com/site/about/guidelines.xhtml

Discussion

Our findings reveal a major reduction in HRQOL for people living with musculoskeletal disorders as identified through REMS screening in comparison to those without musculoskeletal in the Hai district of Tanzania. A good quality of life refers to a person's ability to look after themselves, get around their community, participate in their usual activities, and avoid pain and distress. Many musculoskeletal disorders such as rheumatoid arthritis disproportionately affect women[27]. Here, we find those presenting with musculoskeletal conditions to be predominantly female, and to have a significant reduction in HRQOL scores, which declines further with age. It is well documented that musculoskeletal disorders incur considerable costs to society through lost productivity [28], and in this study 'not working'(anymore) is associated with a statistically significantly negative impact on HRQOL too. Comorbidity was also higher among REMS+ participants and was largely due to their greater prevalence of hypertension. Inflammatory joint diseases and gout are linked to a greater prevalence of cardiovascular disease and hypertension is often the first and most readily observed feature of this tendency[29].

REMS is designed to define the cause of pain or disability in a specific region of the body. This instrument can identify and differentiate between inflammatory joint disorders, such as rheumatoid arthritis, and more common mechanical conditions (such as back pain or osteoarthritis). Furthermore, it can distinguish different types of inflammatory disorders such as psoriatic arthritis from rheumatoid arthritis, although additional radiological or blood tests may be needed to confirm these findings. The country's health care policy should focus on those disorders which cause the greatest loss of independence and for which intervention is affordable and effective. Figure 1 shows how each of the different dimensions of HRQOL – pain, mobility, usual activities, self-care - are negatively impacted in those identified positive as having a musculoskeletal disorder compared with matched controls. Given that low back pain is a common disability and is eminently treatable, resources to support and treat people with this ailment should be made more widely available.

Although our study was designed with the intention of capturing the impact of both musculoskeletal disorders and any associated comorbid conditions, there was the potential for certain comorbidities to be overlooked. Most commonly, these would relate to conditions for which few symptoms would be evident such as renal disease which is a not uncommon association of gout and autoimmune rheumatic diseases. That they are also more likely to have other conditions has led researchers to study associated patterns of multimorbidity in those with arthritis[30]. The reduction of 0.22 utilities in HRQOL found in our study between those screened positive for musculoskeletal disorders and those who did not is of similar magnitude to the reduction in quality of life for people with three or more longstanding conditions living in areas of high socio-economic deprivation in Scotland compared to the general population[31]. In that study, Lawson et al observed a similar utility decrement of 0.21. The fact that the drop in utility found due to musculoskeletal disorders is comparable to that of people living with multiple long-term conditions highlights the importance of musculoskeletal health for the quality of life of patients.

Evidence from other countries shows a wide range of utility reductions in those with rheumatoid arthritis. For example, Hoshi et al [32] find studies range from utility scores of 0.49 to 0.75, likely due to differences in patient populations and study designs, with more recent studies having higher HRQOL values, likely reflecting improved management of patients over the last decade. A systematic review of HRQOL scores in rheumatoid arthritis from Asia found those with moderate to high disease activity scores had EQ-5D utilities of 0.53 and 0.47 respectively which were positively associated with age [33]. However, utility values significantly differed between the countries in which studies were conducted,

For peer review only - http://bmjopen.bmj.com/site/about/guidelines.xhtml

including India averaging 0.40, Taiwan 0.67 and Thailand 0.81. They also found EQ-5D utility values significantly differed between study designs, with EQ-5D values for case-control of 0.65 (0.49-0.80), cohort studies 0.60 (0.54-0.66) and cross-sectional studies 0.72 (0.68-0.75)[33]. The use of other HRQOL tools to generate utilities may also produce variation. Uhlig et al find found a utility reduction using the Short Form Survey 36 of 0.20 in patients aged 50–60 years with musculoskeletal conditions compared to the general population in Norway [34].

Different methodologies to produce value sets may also account in part for variation in results [35]. A limitation of this study is that Tanzania has no value set for utilities. Instead, we used the weights generated in neighbouring Uganda to value health profiles. We have, for this reason, undertaken a sensitivity analysis using the value sets from Ethiopia, another East African country. Although each country employed a combination of slightly different methods, both value sets included the use of time trade-off to generate weights to health profiles where respondents choose between living in a certain impaired health state for a certain length of time, or in full health for a shorter time period [36]. In the Ugandan value set, 'pain and discomfort' was the most important dimension with a decrement of 0.798 for the most extreme level. In Ethiopia, 'pain and discomfort' was the second highest contributor to HRQOL reductions, but with a relatively smaller of decrement of 0.406. Musculoskeletal disorders include a range of different conditions and affect people differently, with pain being the most commonly reported symptom, albeit highly subjective and variable. In studies exploring HRQOL across multiple chronic diseases, pain has been found to have the largest negative impact on HRQOL [37] with musculoskeletal disorders associated with the largest losses of HRQOL[38]. Carreño et al [39] found 'pain or discomfort' to be the highest scoring dimension, with a maximum score in over 12.3% of cases. Our study shows a similar trend with the highest number of extreme scores found in the 'pain or discomfort' and 'mobility' domains equally. Given 'pain or discomfort' is also the most important dimension in the value set used [18], this dimension is driving utility values on both fronts. With such a baseline figure, any future interventions aimed at alleviating symptoms including pain, or indeed any preventive interventions, will likely improve this HRQOL profile.

The dimension 'anxiety and depression' is another area of notable contrast between the two countries' value sets. In Uganda 'anxiety and depression' was found to be the least valued on health. In contrast, in Ethiopia 'anxiety and depression' was rated the most valued. The authors of the Ugandan study note that in Ugandan culture, it is generally considered a weakness for someone to show signs of mental illness or even the need for mental healthcare[18]. Indeed, local clinicians in Tanzania have found while collecting HRQOL data in patients that no-one will score high (severely, extremely impacted) for 'anxiety and depression' given the stigma associated with either recognising or talking about such issues publicly [40]. The fact that mental health is traditionally 'taboo' to talk about in some cultures and religions, is likely to affect the scoring for the 'anxiety and depression' dimension in countries such as Tanzania. Whilst the overall population norms in our study approximate the utility ranges for corresponding age groups from a range of other countries [41], it is notable that our controls have a consistently higher utility score across age bands than might otherwise have been expected, especially in the older age groups. In our study, 'anxiety and depression' had the least important scores, and it is likely that utility values are therefore over-estimated in this population [42]. That cultural norms may influence a person's willingness to disclose their difficulties is a common limitation of many international studies and ours is no exception. Hence, the true burden of disease may be greater than we have reported.

Good practice would dictate that when analysing international studies, country specific value sets should be used to generate utilities valid for that country. In various country comparisons, systematic differences have been found as well as discrepancies when analysing changes from one health state

For peer review only - http://bmjopen.bmj.com/site/about/guidelines.xhtml

to another health state, not just in terms of magnitude but even in the direction of the change [35]. Within these East Africa value sets, generated using similar methods, it is more likely that cultural and contextual reasons are driving a variance in results. In Uganda, the mean for the study sample generating the value set was 0.86 compared with 0.94 in Ethiopia. The authors of the Ethiopian study note evidence of clustering of values at 1 with non-trading of time for milder health states[17]. This is evident in the distribution of utilities (see Supplementary File 2). Overall, compared to the Ethiopian value set, the extreme health states had lower values in Uganda. To overcome these limitations and as part of our wider NIHR study, there is ongoing research to develop an African specific measure of health and wellbeing. This measure will be based on the capability approach [43], a multidimensional approach to wellbeing, to understand what aspects of life are important to people in Tanzania using values from people in Tanzania. Arthritis has potential to affect many aspects of the capabilities that are important to people. When completed, this measure can then be used to provide information to decision-makers about which health and care interventions will most benefit people's lives in Tanzania.

Despite these methodological limitations and varying magnitude of results, it is evident that the impact of musculoskeletal conditions on HRQOL is considerable. Decision makers need information on both costs associated with a condition and the health improvements that can be achieved by intervening for those affected by musculoskeletal disorders when planning for cost effective services. Working across different disciplines, and in both clinical and community settings, our wider NIHR research project has estimated the prevalence of musculoskeletal disorders, its economic burden and impact on HRQOL [44]. In doing so, we provide an initial evidence-base to justify the need to plan effective and cost-effective interventions for the prevention and management of arthritis and musculoskeletal conditions in Tanzania and wider Sub-Saharan Africa.

Conclusion

This study quantifies the significant HRQOL burden caused by musculoskeletal disorders in Kilimanjaro, and likely wider Tanzania. This is the first powered study of its kind in musculoskeletal disorders in Tanzania (and likely wider SSA) using valid screening tools along with estimates of preference-based quality of life. The results will be used to guide clinical health care delivery, interventions design, service provisions, and health promotion and awareness activities both at institutional level, Kilimanjaro Region and national level. The findings from this study will inform those leading the NCD strategies in Tanzania of the magnitude of the impact of arthritis and related musculoskeletal conditions to plan properly for the management of these disorders, develop bespoke interventions to prevent and reduce the impact of these conditions and to improve quality of life for those who live with and suffer from them.

Funding
This study/project was funded by the UK's NIHR Global Health Research Groups Programme (Award no: 17/63/35 https://fundingawards.nihr.ac.uk/award/17/63/35). The views expressed are those of the author(s) and not necessarily those of the NIHR or the Department of Health and Social Care.

Data sharing

MD and SJK have accessed and checked the underlying data. The data that support the findings of this study are available upon reasonable request.

Competing interests None declared.

Author contributions

For peer review only - http://bmjopen.bmj.com/site/about/guidelines.xhtml

Conception and design of the study: BTM, EM

Data acquisition: SJK, NMY, SMB, JH, RW, BTM

Data analysis: EG, MD, SJK, PH

Data interpretation: EG, MD, SJK, JH, CK, SS, RW, NMY, RK, EM

Drafting of the first version of the manuscript: EG

Critical review of the manuscript: All

All authors read and approved the final manuscript.

EG is the guarantor for this article.

Acknowledgements

The NIHR Research Group comprises (in alphabetical order): Sanjura Biswaro, Christopher Bunn, Edith Chikumbu, Jo Coast, Mia Crampin, Manuela Deidda, Nateiya M Yongolo, Eleanor Grieve, Jo Halliday, Victor Katiti, Clive Kelly, Kajiru Kilonzo, Emma Laurie, Emma McIntosh, Blandina Theophil Mmbaga, Gloria Moshi, Elizabeth Msoka, Febronia Shirima, Stefan Siebert, Jennika Virhia, Richard Walker and Sally Wyke. We are grateful for all the support received for this research, including that from our study participants.

For peer review only

For peer review only - http://bmjopen.bmj.com/site/about/guidelines.xhtml

References

1. Eden S, Renaud F, Flora S, et al. The world-wide burden of musculoskeletal diseases: a systematic analysis of the World Health Organization Burden of Diseases Database. *Annals of the Rheumatic Diseases* 2019;78(6):844. doi: 10.1136/annrheumdis-2019-215142
2. Briggs AM, Cross MJ, Hoy DG, et al. Musculoskeletal Health Conditions Represent a Global Threat to Healthy Aging: A Report for the 2015 World Health Organization World Report on Ageing and Health. *Gerontologist* 2016;56 Suppl 2:S243-55. doi: 10.1093/geront/gnw002
3. Koo M, Lu MC. Rheumatic Diseases: New Progress in Clinical Research and Pathogenesis. *Medicina (Kaunas)* 2023;59(9) doi: 10.3390/medicina59091581 [published Online First: 20230831]
4. Arthritis as a potential barrier to physical activity among adults with obesity--United States, 2007 and 2009. *MMWR Morb Mortal Wkly Rep* 2011;60(19):614-8. [published Online First: 2011/05/21]
5. Klinedinst NJ, Resnick B, Yerges-Armstrong LM, et al. The Interplay of Genetics, Behavior, and Pain with Depressive Symptoms in the Elderly. *Gerontologist* 2015;55 Suppl 1(Suppl 1):S67-77. doi: 10.1093/geront/gnv015 [published Online First: 2015/06/10]
6. Blyth FM, Noguchi N. Chronic musculoskeletal pain and its impact on older people. *Best Pract Res Clin Rheumatol* 2017;31(2):160-68. doi: 10.1016/j.berh.2017.10.004 [published Online First: 2017/12/12]
7. Hoy D, Geere JA, Davatchi F, et al. A time for action: Opportunities for preventing the growing burden and disability from musculoskeletal conditions in low- and middle-income countries. *Best Pract Res Clin Rheumatol* 2014;28(3):377-93. doi: 10.1016/j.berh.2014.07.006 [published Online First: 20141022]
8. Storheim K, Zwart JA. Musculoskeletal disorders and the Global Burden of Disease study. *Ann Rheum Dis* 2014;73(6):949-50. doi: 10.1136/annrheumdis-2014-205327
9. Ngowi JE, Munishi C, Ndumwa HP, et al. Efforts to Address the Burden of Non-Communicable Diseases Need Local Evidence and Shared Lessons from High-Burden Countries. *Annals of Global Health* 2023
10. Global, regional, and national incidence, prevalence, and years lived with disability for 328 diseases and injuries for 195 countries, 1990-2016: a systematic analysis for the Global Burden of Disease Study 2016. *Lancet* 2017;390(10100):1211-59. doi: 10.1016/s0140-6736(17)32154-2 [published Online First: 2017/09/19]
11. Safiri S, Kolahi AA, Cross M, et al. Prevalence, Deaths, and Disability-Adjusted Life Years Due to Musculoskeletal Disorders for 195 Countries and Territories 1990-2017. *Arthritis Rheumatol* 2021;73(4):702-14. doi: 10.1002/art.41571 [published Online First: 20210222]
12. Doherty M, Dacre J, Dieppe P, et al. The 'GALS' locomotor screen. *Ann Rheum Dis* 1992;51(10):1165-9. doi: 10.1136/ard.51.10.1165 [published Online First: 1992/10/01]
13. Coady D, Walker D, Kay L. Regional Examination of the Musculoskeletal System (REMS): a core set of clinical skills for medical students. *Rheumatology (Oxford)* 2004;43(5):633-9. doi: 10.1093/rheumatology/keh138 [published Online First: 2004/04/01]
14. United Nations. Designing Household Survey Samples: United Nations 2008.
15. Kilonzo K, Krauth S, Halliday J, et al. Estimating the prevalence, quality of life, economic and societal impact of arthritis in Tanzania: Protocol for a mixed methods study. protocols.io https://dx.doi.org/10.17504/protocols.io.8epv5j8r4l1b/v1, 2023.
16. Feng YS, Kohlmann T, Janssen MF, et al. Psychometric properties of the EQ-5D-5L: a systematic review of the literature. *Qual Life Res* 2021;30(3):647-73. doi: 10.1007/s11136-020-02688-y [published Online First: 2020/12/08]
17. Welie AG, Gebretekle GB, Stolk E, et al. Valuing Health State: An EQ-5D-5L Value Set for Ethiopians. *Value in Health Regional Issues* 2020;22:7-14. doi: https://doi.org/10.1016/j.vhri.2019.08.475
18. Yang F, Katumba KR, Roudijk B, et al. Developing the EQ-5D-5L Value Set for Uganda Using the 'Lite' Protocol. *Pharmacoeconomics* 2022;40(3):309-21. doi: 10.1007/s40273-021-01101-x [published Online First: 2021/11/30]

For peer review only - http://bmjopen.bmj.com/site/about/guidelines.xhtml

19. Ratcliffe J, Huynh E, Stevens K, et al. Nothing About Us Without Us? A Comparison of Adolescent and Adult Health-State Values for the Child Health Utility-9D Using Profile Case Best–Worst Scaling. *Health Economics* 2016;25(4):486-96. doi: https://doi.org/10.1002/hec.3165
20. Pearce N. Analysis of matched case-control studies. *BMJ* 2016;352:i969. doi: 10.1136/bmj.i969
21. White IR, Royston P, Wood AM. Multiple imputation using chained equations: Issues and guidance for practice. *Stat Med* 2011;30(4):377-99. doi: 10.1002/sim.4067 [published Online First: 20101130]
22. Azur MJ, Stuart EA, Frangakis C, et al. Multiple imputation by chained equations: what is it and how does it work? *Int J Methods Psychiatr Res* 2011;20(1):40-9. doi: 10.1002/mpr.329 [published Online First: 2011/04/19]
23. Laurie E, Virhia J, Yongolo N, et al. Activating Equitable Engagement: from research to policy (and back again): a report on dissemination activities for 'NIHR Global Health Research Group on estimating the prevalence, quality of life, economic and societal impact of arthritis in Tanzania. In: Team NGH, ed. unpublished: https://eprints.gla.ac.uk/298157/, 2023.
24. Yongolo NM, Halliday J, Bunn C, et al. Estimating the prevalence and predictors of musculoskeletal disorders in Tanzania: a cross-sectional pilot study. *Pan Afr Med J* 2024;47:36. doi: 10.11604/pamj.2024.47.36.38258 [published Online First: 20240130]
25. Msoka EF, Bunn C, Msoka P, et al. Rapid ethnographic appraisal of community concepts of and responses to joint pain in Kilimanjaro, Tanzania. *BMJ Glob Health* 2024;9(1) doi: 10.1136/bmjgh-2023-013245 [published Online First: 20240131]
26. Devlin N, Finch AP, Parkin D. Guidance to Users of EQ-5D-5L Value Sets. In: Devlin N, Roudijk B, Ludwig K, eds. Value Sets for EQ-5D-5L: A Compendium, Comparative Review & User Guide. Cham: Springer International Publishing 2022:213-33.
27. Angum F, Khan T, Kaler J, et al. The Prevalence of Autoimmune Disorders in Women: A Narrative Review. *Cureus* 2020;12(5):e8094. doi: 10.7759/cureus.8094 [published Online First: 2020/06/17]
28. Hsieh P-H, Wu O, Geue C, et al. Economic burden of rheumatoid arthritis: a systematic review of literature in biologic era. *Annals of the Rheumatic Diseases* 2020;79(6):771. doi: 10.1136/annrheumdis-2019-216243
29. Liang X, Chou OHI, Cheung CL, et al. Is hypertension associated with arthritis? The United States national health and nutrition examination survey 1999-2018. *Ann Med* 2022;54(1):1767-75. doi: 10.1080/07853890.2022.2089911
30. McLoone P, Jani BD, Siebert S, et al. Classification of long-term condition patterns in rheumatoid arthritis and associations with adverse health events: a UK Biobank cohort study. *J Multimorb Comorb* 2023;13:26335565221148616. doi: 10.1177/26335565221148616 [published Online First: 2023/02/18]
31. Lawson KD, Mercer SW, Wyke S, et al. Double trouble: the impact of multimorbidity and deprivation on preference-weighted health related quality of life a cross sectional analysis of the Scottish Health Survey. *Int J Equity Health* 2013;12:67. doi: 10.1186/1475-9276-12-67 [published Online First: 2013/08/22]
32. Hoshi D, Tanaka E, Igarashi A, et al. Profiles of EQ-5D utility scores in the daily practice of Japanese patients with rheumatoid arthritis; Analysis of the IORRA database. *Mod Rheumatol* 2016;26(1):40-5. doi: 10.3109/14397595.2015.1059983 [published Online First: 2015/06/09]
33. Haridoss M, Bagepally BS, Natarajan M. Health-related quality of life in rheumatoid arthritis: Systematic review and meta-analysis of EuroQoL (EQ-5D) utility scores from Asia. *Int J Rheum Dis* 2021;24(3):314-26. doi: 10.1111/1756-185x.14066 [published Online First: 2021/01/25]
34. Uhlig T, Loge JH, Kristiansen IS, et al. Quantification of reduced health-related quality of life in patients with rheumatoid arthritis compared to the general population. *J Rheumatol* 2007;34(6):1241-7. [published Online First: 2007/05/23]

For peer review only - http://bmjopen.bmj.com/site/about/guidelines.xhtml

35. Gerlinger C, Bamber L, Leverkus F, et al. Comparing the EQ-5D-5L utility index based on value sets of different countries: impact on the interpretation of clinical study results. *BMC Research Notes* 2019;12(1):18. doi: 10.1186/s13104-019-4067-9
36. Lugnér AK, Krabbe PFM. An overview of the time trade-off method: concept, foundation, and the evaluation of distorting factors in putting a value on health. *Expert review of pharmacoeconomics & outcomes research* 2020;20(4):331-42. doi: 10.1080/14737167.2020.1779062 [published Online First: 2020/06/20]
37. Wu M, Brazier JE, Kearns B, et al. Examining the impact of 11 long-standing health conditions on health-related quality of life using the EQ-5D in a general population sample. *The European journal of health economics : HEPAC : health economics in prevention and care* 2015;16(2):141-51. doi: 10.1007/s10198-013-0559-z [published Online First: 2014/01/11]
38. Saarni SI, Härkänen T, Sintonen H, et al. The impact of 29 chronic conditions on health-related quality of life: a general population survey in Finland using 15D and EQ-5D. *Qual Life Res* 2006;15(8):1403-14. doi: 10.1007/s11136-006-0020-1 [published Online First: 2006/09/09]
39. Carreño A, Fernández I, Badia X, et al. Using HAQ-DI to estimate HUI-3 and EQ-5D utility values for patients with rheumatoid arthritis in Spain. *Value Health* 2011;14(1):192-200. doi: 10.1016/j.jval.2010.11.001 [published Online First: 2011/01/08]
40. NIHR Global Health Group. 2023.
41. Janssen MF, Szende A, Cabases J, et al. Population norms for the EQ-5D-3L: a cross-country analysis of population surveys for 20 countries. *Eur J Health Econ* 2019;20(2):205-16. doi: 10.1007/s10198-018-0955-5 [published Online First: 2018/02/16]
42. Lugata S, Elinisa M, Doshi B, et al. Symptoms and predictors of depression among university students in the Kilimanjaro region of Tanzania: a cross-sectional study. *Journal of Mental Health* 2021;30(2):255-62. doi: 10.1080/09638237.2020.1793129
43. Coast J, Kinghorn P, Mitchell P. The development of capability measures in health economics: opportunities, challenges and progress. *Patient* 2015;8(2):119-26. doi: 10.1007/s40271-014-0080-1 [published Online First: 2014/07/31]
44. Deidda M, Grieve E, Krauth S, et al. Economic burden of musculoskeletal disorders in Tanzania: results from a community-based survey. *BMJ Open* 2025;15(1):e087425. doi: 10.1136/bmjopen-2024-087425 [published Online First: 20250115]

For peer review only

For peer review only - http://bmjopen.bmj.com/site/about/guidelines.xhtml

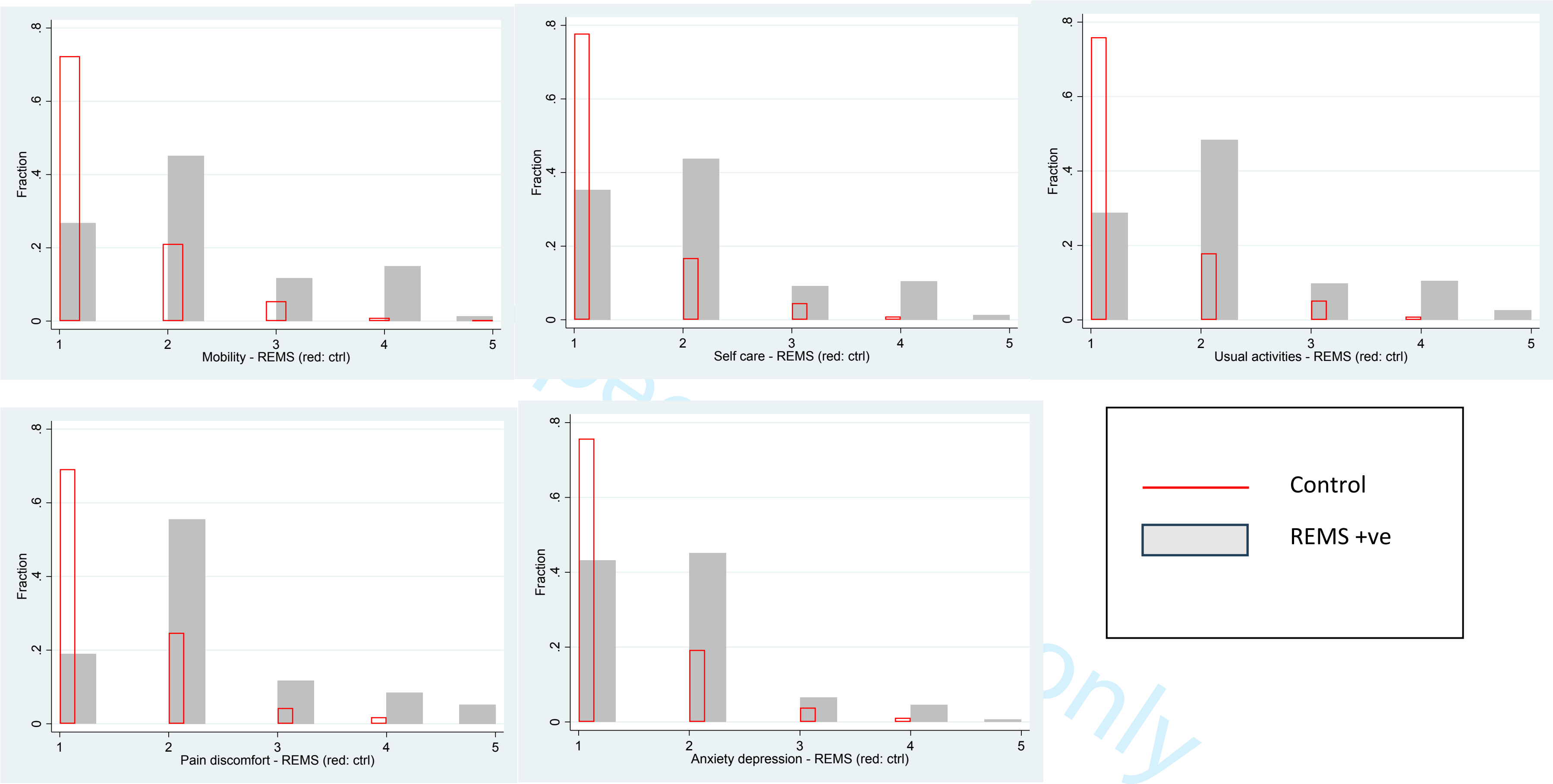

Figure 1: Score distributions for those identified positive as having a musculoskeletal disorder compared with matched controls for each HRQOL dimension (Ugandan tariff)

Supplementary 1: Model fit tests

GLMDIAGL: This program runs the modified Park test, Pearson correlation test, Pregibon link test, and modified Hosmer and Lemeshow test, for a set of power links that range between -2 and 2 in intervals of 0.1. It records the results of these tests as well as the log likelihood, AIC, BIC statistics from each power model. Saved statistics are for the model that uses the family specified by the modified Park test. [glmdiagl "glm QoLUg i.rems_ctrl age i.sex i.religion i.edusimple i.labour i.marrydummy i.familypain i.intsmoke i.intdrink i.everpregnant i.diabetest3 i.hypertensiont3 i.MM, vce(cluster clustervar)"]

```
                ________________ Summary Score Recommendations________________
                 3-(sum p)        sum abs|log(p)|   3-(sum p^.5)      sum(1-p)^2
Ranking         Link    Family    Link    Family    Link    Family    Link    Family

Preferred       1.00       0     -2.00       0      1.00       0      1.00       0
2nd best        0.90       0     -1.90       0      0.90       0      0.90       0
3rd best        0.80       0     -1.80       0      0.80       0      0.80       0

See r(linkmat1), r(linkmat2), and r(familymat) for more detailed results

Family Coding (for r(familymat)

0 = gaussian; 1 = poisson; 2 = gamma; 3 =igaussian; 4 = Preferred family switches
''Conflicting families'': 40 = gaussian; 41 = poisson; 42 = gamma; 43 = igaussian
''Initial values infeasible'': 70 = gaussian; 71 = poisson; 72 = gamma; 73 = igaussian;
5 = None
```

Source: https://drhenryglick.com/identifying-better-links-and-families/

```
 FITTED MODEL:  Link = Power  1; Family =  Gaussian

 Results, Modified Park Test (for Family)

    Coefficient:   -2.60235

    Family, Chi2, and p-value in descending order of likelihood

      Family                            Chi2         P-value

      Gaussian NLLS:                 86.6945          0.0000
      Poisson:                      166.1237          0.0000
      Gamma:                        271.1560          0.0000
      Inverse Gaussian or Wald:     401.7912          0.0000


 Results of tests of GLM Power  1 link

      Pearson Correlation Test:                       1.0000
      Pregibon Link Test:                             0.0006
      Modified Hosmer and Lemeshow:                   0.0000
      SS: sqr1_p                                      1.9988
      SS: sqrtp                                       1.9755
      SS: logp                                       32.7470
      SS: sump                                        1.9994
      R-square                                        0.3478
      Log likelihood                                -52.9043
      AIC                                             0.2865
      BIC                                         -2880.1483
      Deviance                                       35.4903


 **Gaussian family given identity link
```

For peer review only - http://bmjopen.bmj.com/site/about/guidelines.xhtml

Supplementary 2: Distributions of Utilities

Distribution of utilities using Ugandan and Ethiopian tariffs: population

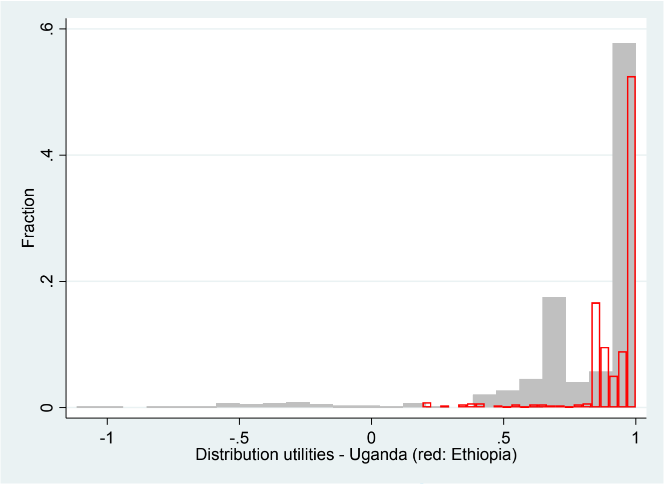

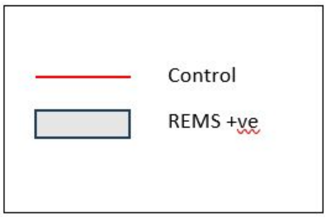

Distribution of utilities using Ugandan and Ethiopian tariffs: REMS positive

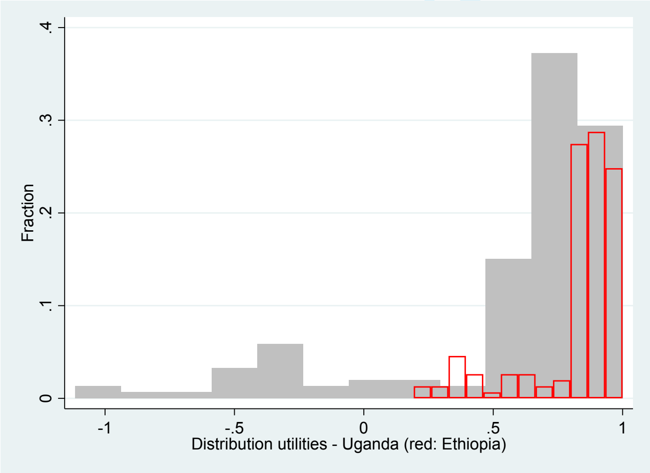

Distribution of utilities using Ugandan and Ethiopian tariffs: Controls

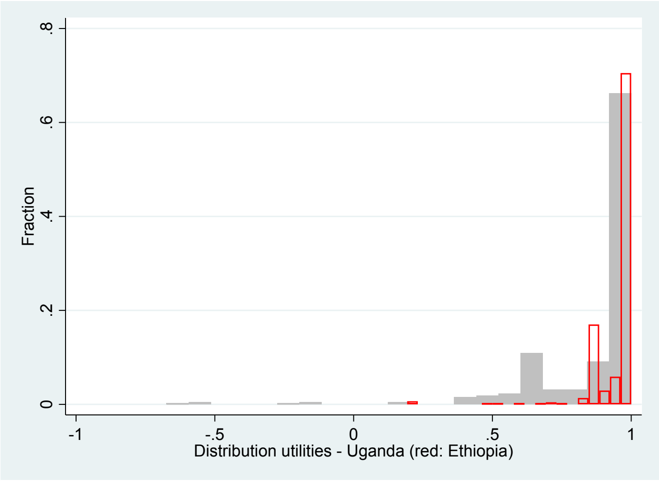

For peer review only - http://bmjopen.bmj.com/site/about/guidelines.xhtml
